# Characterization of two novel species of the genus *Flagellimonas* reveals the key role of vertical inheritance in the evolution of alginate utilization loci

**DOI:** 10.1128/spectrum.00917-25

**Published:** 2025-07-07

**Authors:** Juan Yu, Jia-Wei Gao, Ke Cao, Dong-Yan He, Lin Xu, Ge-Yi Fu, Cong Sun

**Affiliations:** 1 College of Life Sciences and Medicine, Zhejiang Sci-Tech University 12646, Hangzhou, China; 2 Zhejiang Engineering Research Center for the Development Technology of Medicinal and Edible Homologous Health Food, Shaoxing Biomedical Research Institute of Zhejiang Sci-Tech University Co., Ltd, Shaoxing, China; 3 Key Laboratory of Marine Ecosystem Dynamics, Ministry of Natural Resources & Second Institute of Oceanography, Ministry of Natural Resources 118476, Hangzhou, China; University of Maryland Baltimore County, Baltimore, Maryland, USA

**Keywords:** *Flavobacteriaceae*, *Flagellimonas*, alginate utilization loci, CAZyme, vertical inheritance

## Abstract

*Flavobacteriaceae* is the major participant in the degradation of algal polysaccharides. With diverse polysaccharide utilization loci (PULs) and specific carbohydrate-active enzymes (CAZymes), *Flavobacteriaceae* strains appear to have different abilities in algal polysaccharide degradation and therefore change their roles in the bacterial community. Here, we identified two novel isolates as two novel species of genus *Flagellimonas* with the names *Flagellimonas alginolytica* sp. nov. and *Flagellimonas cixiensis* sp. nov. Furthermore, the comprehensive genomic comparison of 41 *Flagellimonas* genomes revealed that *Flagellimonas* strains were diverse in the CAZymes and PUL profiles and exhibited a preference for polysaccharides derived from brown algae. The evolutionary analysis of alginate utilization loci (AUL) in this genus illuminated that the function genes in AULs, that is, PL7 and PL17, were more reliant on the stable inheritance from ancestors associated with gene duplication and loss rather than horizontal gene transfer (HGT) from outside, and the AUL structures exhibited a trend of simplification which resulted in the incidental decrease in alginate degradation ability. This study highlights the important role of vertical inheritance in the evolution of AULs and proves that the discrepancy in AUL structure can arouse phenotypic differences, providing a new perspective on the evolution of AUL and the niche adaptation mechanism of *Flavobacteriaceae* strains.

**IMPORTANCE:**

*Flavobacteriaceae* play an important role in the marine carbon cycle with their noteworthy ability in algal polysaccharides degradation, which is primarily reliant on diverse polysaccharide utilization loci (PULs). Our study highlights the crucial role of vertical inheritance in the evolution of alginate utilization loci (AUL) in *Flagellimonas* strains and reveals the AUL structural simplification found in *Flagellimonas* strains that will lead to the reduction of alginate degradation ability. These insights advance understanding of niche adaptation strategy and related evolutionary mechanisms of *Flavobacteriaceae* strains.

## INTRODUCTION

Bacteroidota is considered a major part of bacterial communities in the marine environment and plays an important role, participating in polysaccharide degradation, nitrogen cycling, and other functions (1). *Flavobacteriaceae*, one of the most important and largest components of this phylum, contains more than 160 validly published genera (2). The genus *Flagellimonas*, a member of the family *Flavobacteriaceae* of the phylum Bacteroidota, was first identified by Bae et al. in 2007 with *Flagellimonas eckloniae* as the type species (3). At the time of writing (January 2025), the genus *Flagellimonas* comprised 47 validly published species (https://lpsn.dsmz.de/genus/Flagellimonas) (4). Most members of the genus *Flagellimonas* were isolated from salty habitats such as tidal flats (5), salt lakes (6), marine sediment (7, 8), marine invertebrates (9), seawater (4, 10), and mangrove sediments (11). Members of the genus *Flagellimonas* are Gram-stain­-negative, facultatively anaerobic or strictly aerobic, have menaquinone-6 (MK-6) as the major respiratory quinone, and usually contain phosphatidylethanolamine (PE) as the main polar lipid and iso-C_15:0_ as the predominant fatty acid (6, 8).

The degradation of polysaccharides, an important ability of *Flavobacteriaceae*, is dependent on diverse carbohydrate-active enzymes (CAZymes) (12). Glycoside hydrolase (GH), polysaccharide lyase (PL), and carbohydrate-binding module (CBM) are considered the major function classes for polysaccharide degradation (13, 14). Polysaccharide utilization loci (PULs) are specific gene clusters, mainly found in Bacteroidota, consisting of proteins such as closely related CAZymes, SusC and SusD-like proteins that sense, bind, transport, and hydrolyze polysaccharides for the highly efficient degradation or utilization of polysaccharides (15, 16). The SusC-like protein is a TonB-dependent receptor/transporter (TBDR), and the SusD-like protein is the outer membrane sugar-binding protein; they usually function together to take up degraded polysaccharides (oligosaccharides) into the cell and are located nearby as SusC/D pairs in PULs (17, 18). The auxiliary genes in PULs are partly or totally comprised of transcriptional regulators, sulfatases, monosaccharide transporters, and enzymes (19). Since the substrates of most PULs are specific and can be predicted by the profiles of CAZymes, PULs are widely used in the function prediction about polysaccharides degradation of strains, whether in gut or marine environments (15, 20).

Algae, as the most important primary producers in marine ecosystems, produce large quantities of polysaccharides as their structural or storage compounds (21). Brown algae, such as *Laminaria japonica*, are widely distributed in coastal regions and contain alginate as one of the major polysaccharides (about 40% of the dry weight of brown algae) (22). Alginate consists of two uronic acid units, that is, β-D-mannuronate (M) and α-L-guluronate (G), randomly linked with β−1,4-glycosidic bonds. Alginate lyase can break the link through a β-elimination reaction (23). According to the classification in the Carbohydrate-Active enZYmes (CAZy) database, alginate lyases are assigned to 15 PL families, including PL5, PL6, PL7, PL8, PL14, PL15, PL17, PL18, PL31, PL32, PL34, PL36, PL38, PL39, and PL41 (24, 25). Alginate-related PUL, also known as the Alginate Utilization Loci (AUL), typically consists of at least one alginate lyase and one SusC/D pair. Some AULs contain transcriptional regulators such as GntR, and monosaccharide metabolism-related proteins such as phosphorylates 2-keto-3-deoxygluconate (KDGK) and 2-keto-3-deoxy-6-phosphogluconate aldolase (KDPGA) (17, 19).

The first AUL was found in the marine flavobacterium *Zobellia galactanivorans* in 2012, and phylogenetic analysis showed it originated from an ancestral marine flavobacterium and independently transferred to marine proteobacteria (26). Subsequently, AULs were reported in diverse *Flavobacteriaceae* strains. The AUL in *Gramella forsetii* KT0803 is in strong synteny with the AULs in other Bacteroidetes (27). As for genus *Maribacter*, strains containing AULs are located together in one lineage apart from strains encoding fewer PLs (28). In the marine bacterial communities colonizing a mix of alginate and pectin particles, different variants of PLs in AULs are found to be activated by different alginate characteristics (29). Compared with other *Zobellia* strains, *Z. galactanivorans* Dsij^T^ has proven to be the “sharing” pioneer in the degradation of *Laminaria digitate*, suggesting the differences in AULs may affect the ecological niches (30). Besides AULs, the AUL-like alginate utilization system (AUS) is widely explored in other taxa such as *Vibrionaceae*. Three AUS are identified in different regions of the *Vibrio pelagius* WXL662 genome, with alginate lyases exhibiting distinct substrate preferences (31). Hehemann et al. reported that horizontal gene transfer drives adaptive radiation in marine *Vibrionaceae*, leading to specialized roles in alginate degradation: pioneers, harvesters, and scavengers, allowing for fine-scale resource partitioning (32).

In this study, we isolated and identified two novel strains from different samples, proposed them as two novel species of genus *Flagellimonas,* that is, *Flagellimonas alginolytica* sp. nov. and *Flagellimonas cixiensis* sp. nov. Then, we performed a comprehensive genomic comparison of genus *Flagellimonas,* accompanied by the in-depth evolutionary analysis of AULs to reveal the CAZymes and PUL profiles of *Flagellimonas* strains and the detailed evolutionary process of AULs in *Flagellimonas* strains. With this study, we aim to answer two questions: (i) how the diverse AULs appear within genus *Flagellimonas* and (ii) how the differentiation of AULs influences the ecological niche of strains.

## MATERIALS AND METHODS

### Isolation and cultivation, and preservation

Strain C4^T^ was isolated from a seawater mixture sample collected from the Yellow Sea (35°40′ N, 121°00′ E). Strain GZD32^T^ was isolated from tidal flat sediment collected in Cixi, Zhejiang, China (30°10′ N, 121°14′ E). The sample was serially diluted to a concentration of 10^−4^ and spread onto marine agar (MA, BD). After incubation at 28°C for 3 days, the single colonies of strain C4^T^ and strain GZD32^T^ were picked out and routinely incubated on MA at 28°C for phenotypic, biochemical, and chemical analyses. For long-term preservation, the novel strain was stored at −80°C in broth containing 25% (vol/vol) glycerol. For cultivation, all strains were grown in marine broth (MB, BD) at 28°C for 3 days. For comparison, reference strains *Flagellimonas marinaquae* JCM11811^T^ and *Flagellimonas zhangzhouensis* MCCC 1F01096^T^ were obtained from the Japan Collection of Microorganisms (JCM) and the Marine Culture Collection of China (MCCC), respectively.

### Morphological, physiological, biochemical, and chemotaxonomic features

Growth at different temperatures (4, 10, 15, 20, 25, 27, 30, 37, 40, and 45°C) was assessed using MB. To determine the optimal pH and growth range, MB was adjusted to pH 5.0–10.0 (interval of 0.5 pH unit) using appropriate buffers: 2-(N-morpholino) ethanesulfonic acid (pH 5.0–5.5), 3-morpholinopropanesulfonic acid (pH 6.0–7.5), Tricine (pH 8.0-8.5), and N-cyclohexyl-3-aminopropanesulfonic acid (pH 9.0–10.0) (33). NaCl tolerance was tested in NaCl-free MB, which was prepared according to the standard MB formulation but omitted NaCl. NaCl was then added to the medium at concentrations ranging from 0 to 12.0% (wt/vol) with 1.0% increments. All tests were measured at OD_600_ nm using a UV/visible spectrophotometer (UV756RT, Yoke Instrument) and repeated three times.

Hydrolysis of starch, tyrosine, cellulose, Tween 20, 40, 60, and 80, and casein was tested using modified MA. The medium was prepared according to the standard MA formulation, but the concentration of yeast extract and peptone was reduced to 0.1 g/L and 0 g/L, respectively. Related substrates were added to modified MA at the following concentrations: 2 g/L starch, 5 g/L tyrosine, 2 g/L cellulose, 10 g/L casein, and 10 g/L of Tween 20, 40, 60, or 80, respectively (34). API 20NE, API ZYM, and API 50CH strips (bioMérieux) were utilized for analyzing the physiological and biochemical features according to the instruction manuals. The bacterial cultures were centrifuged and resuspended in sterile 2% (wt/vol) NaCl solution, then used as the inoculum for API strips.

The colony morphology of novel isolates was observed 3 days after scribing on MA, and the cells’ morphology was examined by transmission electron microscopy (TEM, JEM-1400Flash) with cells collected after 3 days incubation on MA. The Gram staining method was used to detect the Gram reaction (35). Anaerobic growth was tested as described (36). Catalase activity was assessed by adding 3% hydrogen peroxide to the colony and observing bubble formation. Oxidase activity was determined by adding 1% p-aminodimethylaniline oxalate solution to the colony and checking the change of colony color after one minute (37). For carbohydrate utilization tests, filter-sterilized sugars (0.2% wt/vol), alcohols (0.2% wt/vol), organic acids (0.1% wt/vol), or amino acids (0.1% wt/vol) were added to modified MB medium (absence of yeast extract and peptone), respectively. After inoculation and incubation at 28°C for 3 days, the medium was measured at OD_600_ nm using a UV/visible spectrophotometer (UV756RT, Yoke Instrument).

The cells for fatty acid analysis were harvested after 3 days incubation on MA at 28°C, after saponification, methylation, and extraction, submitted to the Sherlock microbial identification system with standard MIS library software (version 6.5, MIDI) and a gas chromatograph (Agilent 8860) for identification and quantification (34, 38). The polar lipids were extracted according to the described method and analyzed using the two-dimensional thin-layer chromatography (TLC) method with silica gel 60 F_254_ plates (10 × 10 cm; Merck, Germany) (36, 39). Total polar lipids, phospholipids, aminolipids, and glycolipids were extracted and analyzed by two-dimensional thin-layer chromatography (TLC), and visualized using specific staining reagents as described by Xu et al. (2016) and Ying et al. (2021) (40, 41). Respiratory quinones were extracted with a chloroform/methanol (2:1, vol/vol) mixture and identified using an HPLC-MS system (Agilent 1200) following the protocols of Xu et al. (2016) (40).

### Genome sequencing and phylogenetic analyses

Genomic DNA was extracted as described (42) and submitted to Guangdong Magigene Biotechnology Co., Ltd. for genome sequencing. The genome was sequenced using the Illumina NovaSeq 6000 platform (Illumina Inc.), and assembly was done with SPAdes v.3.10.1. The genome quality was assessed using CheckM v.1.0.7 (43). The complete 16S rRNA gene sequences were extracted by RNAmmer v.1.2 (44), and sequence similarities of 16S rRNA gene sequences were compared using the EzBioCloud (https://www.ezbiocloud.net/) (45). The 16S rRNA gene sequences of closely related type strains were downloaded from NCBI and analyzed using the MEGA 11.0 software with the ClustalW program for sequence alignment (46) and neighbor-joining (NJ) (47), maximum-likelihood (ML) (48) methods for the reconstruction of phylogenetic trees. For all methods, Kimura’s two-parameter model (49) and 1,000 replications were employed. *Zeaxanthinibacter aestuarii* S2-22^T^ was used as an outgroup.

For phylogenomic analysis, 39 genomes of type strains in the genus *Flagellimonas* were downloaded from NCBI (https://www.ncbi.nlm.nih.gov). All downloaded genomes, together with genomes of strains C4^T^ and GZD32^T^, were annotated by the RAST server (50). As for the phylogenomic tree, species annotation was first performed using the local version of GTDB-tk (51), which involved three steps: identification, alignment, and classification, then reconstructed using FastTree v2.1.11, with *Zeaxanthinibacter enoshimensis* DSM 18435^T^ used as an outgroup. The values of average nucleotide identity (ANI) and the digital DNA-DNA hybridization (dDDH) were analyzed by OrthoANI Tool version 0.93.1 (52, 53) and Genome-to-Genome Distance Calculator 3.0 web server (#) (45), respectively.

### PUL prediction and evolutionary analysis

All available genomes of type strains of the genus *Flagellimonas* were submitted to the prediction of PUL. First, CAZyme was annotated and assigned into families and subfamilies using dbCAN3 (https://bcb.unl.edu/dbCAN2/blast.php) (54). The CAZymes identified by two out of the three search algorithms in dbCAN3 (HMMER, DIAMOND, and eCAMI) were considered as the CAZymes used in the further study (55, 56). The SusC/D protein sequences were predicted by the RAST annotation system and then confirmed by a search against the Pfam database (http://pfam.xfam.org) for SusC (TIGR04056 and TIGR04057) and SusD (PF07980, PF12741, and PF12771) using BLASTP. Peptidase was annotated against MEROPS (https://www.ebi.ac.uk/merops/) (57) and the local server BLASTP (only considering values e < 1e-15). Sulfatase was identified using SulfAtlas version 2.3.1 (https://sulfatlas.sb-roscoff.fr/sulfatlas/) (58) and the local server BLASTP (only considering values of e < 1e-15). PULs were first predicted based on the PULDB databases (http://www.cazy.org/PULDB/)(59), and the rest of the PULs were also predicted following two categories: (i) at least one pair of SusC/D genes, with at least one GH or PL gene present within 10 genes upstream or downstream of the SusC/D genes; (ii) at least one SusC gene, with ≥5 GH genes located within 10 genes upstream or downstream of the SusC gene. All data were visualized by chiplot (https://www.chiplot.online). The evolutionary events about specific CAZyme in PUL, that is, PL6, PL7, and PL17, were predicted by AnGST with the following parameters: loss = 1.0, duplication = 2.0, HGT = 3.0 (60).

### Enzyme activity assay

Considering the structures of PULs, three *Flagellimonas* strains (*Flagellimonas okinawensis* MCCC 1K08502^T^, Strain C4^T^, *Flagellimonas nanhaiensis* MCCC 1K03557^T^) were selected as the representative strains in the assessment of enzyme activities. The strains were initially cultured in MB until the late logarithmic phase and then transferred to modified MB with 2% inoculum. Modified MB was prepared according to the formula of MB, but the concentration of yeast extract and peptone was reduced to 0.1 g/L and 0 g/L, respectively, and 1 g/L sodium alginate (medium viscosity, Sigma) was used as a carbon source. After 12, 24, and 36 hours of incubation, cell growth (OD_600 nm_) and crude alginate lyase activity were measured. The cultures were centrifuged at 12,000 rpm for 10 minutes to remove the cells. The supernatant was considered the crude alginate lyase. It was determined by measuring the reducing sugar using the 3,5-dinitrosalicylic acid (DNS) colorimetric method (61), and enzyme activity was calculated accordingly (62). One unit (U) of enzyme activity was defined as the amount of enzyme required to release 1 µmol of reducing sugar per min.

To determine the enzymolysis product of the crude alginate lyase, thin-layer chromatography (TLC) was performed. In brief, the 10 mL crude alginate lyase received above was further concentrated using a protein centrifugal ultrafiltration tube (30 kDa MWCO, Merck), then 200 µL of the concentrated solution was mixed with 400 µL sodium alginate solution (0.5%, wt/vol). After incubation at 40°C for 24 h, the reaction was terminated by boiling for 10 min. After centrifugation at 8,000 rpm for 5 min, the supernatant was collected as the enzymolysis product. 5 µL of supernatant was loaded on pro-coated silica gel TLC plates (60 F_254_, Merck) and spread with 1-butanol/acetic acid/water solution (2:1:1, vol/vol/vol). After 15–20 min, the plates were sprayed with sulfuric acid/methanol solution (1:4, vol/vol) and heated at 110°C for 5 min for staining.

## RESULTS AND DISCUSSION

### Phylogenetic analyses based on 16S rRNA gene and genome sequences

The 16S rRNA gene sequence of strain C4^T^ (1,415 bp in length, accession number PP762181) and strain GZD32^T^ (1,384 bp in length, accession number PP762183) were obtained. The 16S rRNA sequence similarity between the two novel strains was 98.1% and the most closely related strain of both strains, C4^T^ and GZD32^T^, was found as *Flagellimonas marinaquae* SW-63^T^ with 98.2% and 98.6% 16S rRNA gene sequence similarity, respectively. For strain C4^T^, the closely related strains were followed by *Flagellimonas* chongwuensis HICW^T^ (98.1%), *Flagellimonas abyssi* W52^T^ (98.0%), and *Flagellimonas oceani* 501str8^T^ (97.8%). However, for strain GZD32^T^, they were *Flagellimonas lutimaris* KCTC 22173^T^ (98.4%), *Flagellimonas zhangzhouensis* 12C25^T^ (98.3%), and *Flagellimonas* alvinocaridis SCR12^T^ (98.3%).

**Fig 1:**
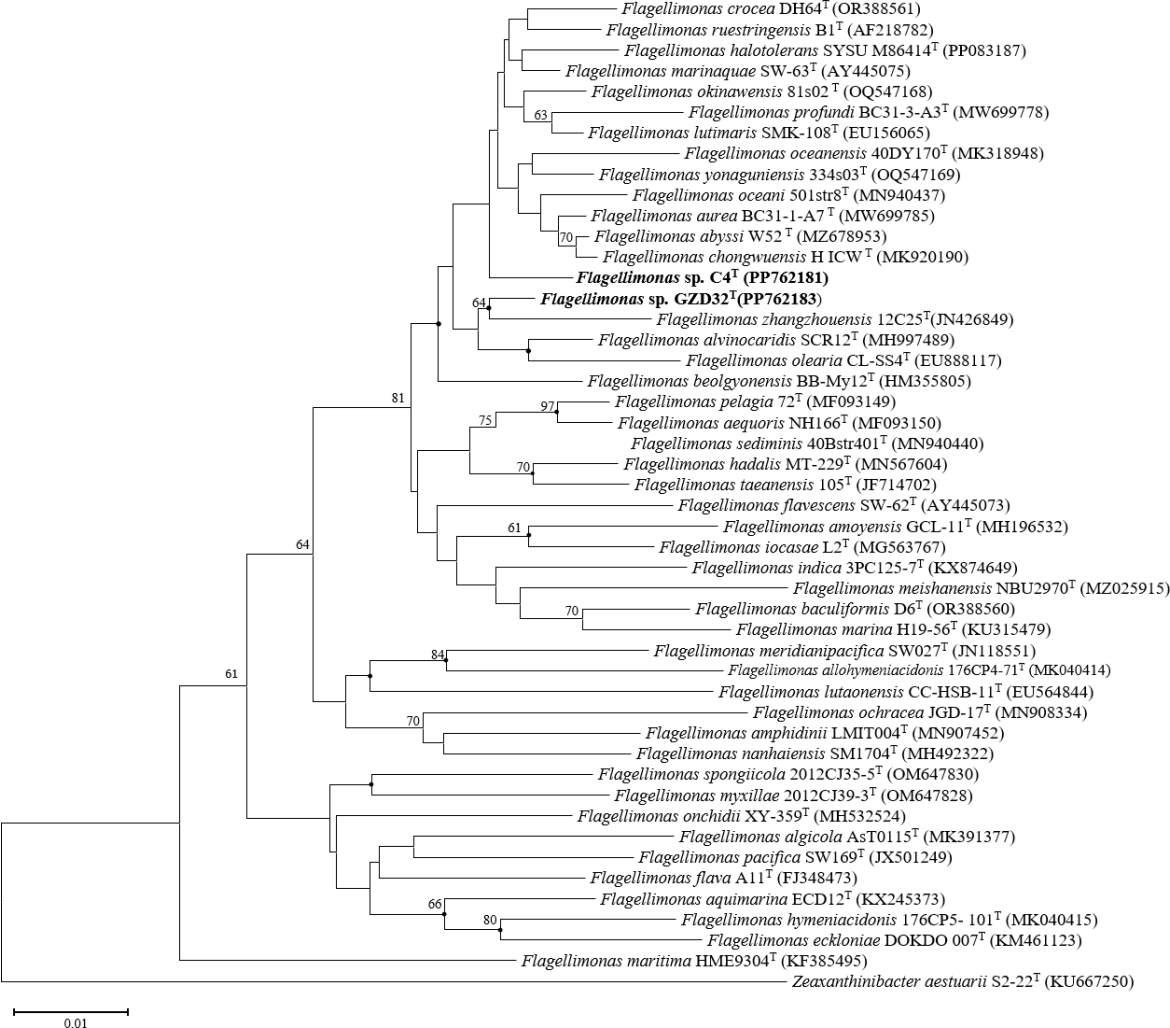
Neighbor-joining phylogenetic tree based on 16S rRNA gene sequences showing the relationships between strains C4^T^, GZD32^T^, and related taxa in genus *Flagellimonas*. Bootstrap values of less than 60% are not shown. Filled circles indicate branches that were recovered with both two methods (neighbor-joining, maximum-likelihood). Bootstrap values are based on 1,000 replicates; Bar, 0.01 substitutions per nucleotide position. *Zeaxanthinibacter aestuarii* S2-22^T^ (KU667250) was used as the outgroup.

The phylogenetic trees, reconstructed using NJ and ML methods, both showed that strain C4^T^ was closely related to *F. marinaquae* SW-63,^T^ and strain GZD32^T^, along with *F. zhangzhouensis* 12C25^T^, formed a distinct branch (Fig. 1). The ANI and dDDH values between strains C4^T^ and GZD32^T^ were 79.2% and 19.6%, respectively. In comparison, the ANI and dDDH values between these two strains and other *Flagellimonas* species ranged from 77.6% to 85.9% and 17.4% to 29.9%, respectively. They are both below the thresholds for species delineation (ANI <95%, dDDH <70%), supporting the classification of the two isolates as two novel species of the genus *Flagellimonas* (Table S1).

The complete genome sequences of strains C4^T^ (CP169638.1) and GZD32^T^ (CP169639.1) both had one chromosome in 3,722,406 bp and 3,387,511 bp length, respectively. The genomic completeness and contamination values for strains C4^T^ and GZD32^T^ were estimated to be 99.4% and 0.7%, and 99.3% and 0.3%, respectively. The DNA G + C content of strains C4^T^ and GZD32^T^ was 41.3% and 40.3%, respectively. The annotation results of the RAST system showed that the genomes of strains C4^T^ and GZD32^T^ contained 3,482 and 3,081 genes, respectively, while both contained 42 RNA genes (Fig. S1).

### Morphological, physiological, biochemical, and chemotaxonomic characteristics

Strains C4^T^ and GZD32^T^ were both Gram-stain-negative, rod-shaped, and facultative anaerobic. The cells of strain C4^T^ were rod-shaped with 2.3–3.2 μm in length and 0.4–0.8 μm in width, and motile with a lateral flagellum. The cells of strain GZD32^T^ were rod-shaped with 1.7–2.6 μm in length and 0.2–0.3 μm in width (Fig. S2). The two strains both formed yellow, round, opaque, border smooth, and convex colonies after being cultivated on MA for 3 days. Strain C4^T^ could grow in 15°C–40°C (optimum 37°C) and pH 5.5–10 (optimum 7.0), with the concentration of NaCl at 0%−10% (wt/vol) (optimum 2%), while strain GZD32^T^ could grow in 18°C−40°C (optimum 28°C) and pH 5.5−8.5 (optimum 7.0), with the concentration of NaCl at 0.5%−8% (wt/vol) (optimum 1.5%). Differing from reference strains (*F. marinaquae* JCM11811^T^, *F. zhangzhouensis* MCCC 1F01096^T^, *F. eckloniae* DOKDO 007^T^), both strains C4ᵀ and GZD32ᵀ were facultative anaerobes. In addition, strain C4ᵀ possessed flagella and exhibited starch hydrolysis activity, but was absent in lipase (C14) and chymotrypsin, and could not hydrolyze Tween 20 or reduce nitrate to nitrogen. While strain GZD32ᵀ was negative for oxidase and chymotrypsin. Detailed physiological and biochemical characteristics are given in Table 1.

**TABLE 1:** Characteristics that differentiate strains C4^T^ and GZD32^T^ from closely related taxa of the genus^*a*^^,^^*b*^

Characteristic	1	2	3	4	5
Flagella	+	−	−	−	+
Temperature range (°C) for growth	15−40 (37)	18−40 (28)	10−44 (30−37)^*c*^	10−40 (25−28)^*d*^	17−36 (26−29)^*e*^
pH range for growth	5.5−10 (7)	5.5−8.5 (7)	5−ND (7)^*c*^	4−11 (7)^*d*^	7−9 (8)^*e*^
NaCl concentration (%) for growth	0−10 (2)	0.5−8 (1.5)	2 (ND−9)^*c*^	ND (2)^*d*^	1.9−5.4 (2.7–3.1)^*e*^
Facultative anaerobic growth	+	+	−	−	−^*f*^
Oxidase	+	−	+	+	−^*f*^
Catalase	+	+	+	+	+^*f*^
Hydrolysis of starch	+	−	−	−	−^*f*^
Tween 20	−	+	+	+	+^*f*^
API 20NE:					
Reduction of nitrate to nitrogen	-	+	+	+	ND
Hydrolysis of gelatin	+	+	−	+	+^*f*^
Enzyme activities (API ZYM)					
Esterase (C4)	+	+	+	+	−^*f*^
Esterase lipase (C8)	+	+	+	+	−^*f*^
Lipase (C14)	−	+	−	+	ND
Chymotrypsin	−	−	+	+	ND
α-galactosidase	+	+	+	+	−^*f*^
β-galactosidase	+	+	+	+	−^*f*^
N-acetyl-β-glucosaminidase	+	+	+	+	−^*f*^
API 50CH					
Glycerol, D-ribose, D-mannitol, and Inulin	+		−	−	ND
L-arabinose and D-xylose, D-melezitose, methyl-αD-mannopyranoside	+	+	−	+	ND
L-rhamnose, D-fucose and L-fucose, D-lyxose	−	−	−	+	ND
Gentiobiose	+	+	+	−	ND
D-tagatose	+	−	−	+	ND
Respiratory quinone	MK-6	MK-6	MK-6	MK-6	MK-6^*f*^
Polar lipids	PE, APG, L (3)	PE, APG, GL, L (2)	PE, L (3)	PE, AGL, L (2)	PE^*f*^
DNA G + C content (%)	41.3	40.3	43.4	39.9	37.8

^*a*^ Strains: 1, Strain C4^T^; 2, Strain GZD32^T^; 3, *Flagellimonas marinaquae* JCM11811^T^; 4, *Flagellimonas zhangzhouensis* MCCC1F01096^T^; 5, *Flagellimonas eckloniae *DOKDO 007^T^ (3, 4).

^*b*^ All data were obtained from this study unless indicated. +, Positive; –, Negative; w, weakly positive; ND, not detected. PE, phosphatidylethanolamine; GL, glycolipids; APG, aminophosphoglycolipid; AGL, aminoglycolipids; L, lipid.

^*c*^ Data from Reference (6).

^*d*^ Data from Reference (11).

^*e*^ Data from Reference (3).

^*f*^ Data from Reference (4).

Both strains C4^T^ and GZD32^T^ contained iso-C_15:0_ (21.6%, 15.8%) and iso-C_17:0_ 3-OH (14.0%, 19.0%) as the major fatty acids (>10%), which were similar to reference strains. In addition, strains C4^T^ and GZD32^T^ contained iso-C_15:1_ G and summed feature 8 (C_18:1_ *ω*6*c* and/or C_18:1_ ω*7c*) as the major fatty acids, respectively. Compared with the reference strains, the presence of summed feature 8 in strains C4^T^ (3.3%) and GZD32^T^ (10.0%) was a distinguishing feature (Table 2). The sole respiratory quinone detected in both strains C4^T^ and GZD32^T^ was MK-6, consistent with other members of the genus *Flagellimonas*. Strains C4^T^ and GZD32^T^ shared phosphatidylethanolamine (PE), aminophosphoglycolipid (APG), and two unidentified lipids (L1, L2) as the major polar lipids; however, strain C4^T^ contained one additional unidentified lipid (L3) and strain GZD32^T^ additionally had one unidentified glycolipid (GL) (Fig. S3). The presence of PE is a common feature of the genus *Flagellimonas,* and the additional APG could distinguish two novel isolates from the reference strains.

**TABLE 2:** Comparison of cellular fatty acid compositions of novel isolate and closely related taxa of the genus *Flagellimonas^a^^,^^b^*

Fatty acids	1	2	3	4	5^*d*^
Straight chain					
C_15:0_	-	-	-	-	3.3
C_16:0_	1.5	2.2	7.7	TR	TR
C_17:0_	TR	TR	3.3	TR	-
Unsaturated					
C_15:1_ ω*5c*	TR	TR	2.0	1.2	-
C_15:1_ ω*6c*	TR	TR	-	1.2	-
C_17:1_ ω*8c*	TR	1.6	TR	TR	-
Hydroxy					
C_17:0_ 2-OH	TR	1.4	TR	-	-
C_17:0_ 3-OH	1.4	-	TR	TR	2.2
iso-C_12:0_ 3-OH	-	2.2	-	-	-
iso-C_15:0_ 3-OH	3.8	3.8	5.4	4.1	8.8
iso-C_16:0_ 3-OH	3.9	TR	3.1	2.7	1.1
iso-C_17:0_ 3-OH	**14.0**	**19.0**	**24.0**	**14.0**	**18.9**
Branched chain					
iso-C_14:0_	3.1	-	1.1	4.3	-
iso-C_16:0_	4.8	TR	1.2	2.9	-
iso-C_17:0_	1.0	TR	TR	1.1	-
iso-C_15:0_	**21.6**	**15.8**	**48.3**	**20.6**	**24.5**
iso-C_15:1_ G	**12.9**	8.4	6.5	**19.7**	**28.4**
anteiso-C_15:0_	2.7	1.1	2.5	2.1	TR
Summed feature 3^*c*^	3.2	5.0	2.2	TR	6.6
Summed feature 8^*c*^	2.6	**10.0**	-	TR	-
Summed feature 9^*c*^	-	3.9	3.3	TR	-

^*a*^ Strains: 1, Strain C4^T^; 2, Strain GZD32^T^; 3, *Flagellimonas marinaquae* JCM11811^T^; 4, *Flagellimonas zhangzhouensis* MCCC 1F01096^T^; 5, *Flagellimonas eckloniae *DOKDO 007^T^ (63).

^*b*^ All data were obtained from this study unless indicated. Values are percentages of total fatty acids. Fatty acids representing >10 % of the total are in bold type. -, not detected. TR, traces (<1.0 %).

^*c*^ Summed feature 3 contains C_16:1_ *ω*7*c* and/or C_16:1_ *ω*6*c*; summed feature 8 contains C_18:1_ *ω*6*c* and/or C_18:1_ *ω*7*c*; summed feature 9 contains iso-C_17:1_ *ω*9*c*.

^*d*^ Data from Reference (63).

### Comparative genomic analyses of metabolic characteristics

The genome size of the genus *Flagellimonas* ranges from 3.2 to 4.5 Mb, with G + C content between 35.6% and 45.7% (Table S2). The genome of *Flagellimonas meishanensis* NBU2970^T^ is the smallest, while the genome of *Flagellimonas oceani* 501str8^T^ is the largest. The G + C content of *Flagellimonas aquimarina* ECD12^T^ is the lowest, while *Flagellimonas amoyensis* GCL-11^T^ has the highest G + C content. Genome expansion has been associated with increased metabolic versatility, enhanced stress response, and improved environmental sensing capabilities (64, 65), while differences in G + C content are often linked to genomic stability and adaptation to environmental factors such as temperature and salinity (66). The large differences in genome size and G + C content among the strains in this genus likely reflect the ecological diversity and adaptability of *Flagellimonas* strains.

We further compared the metabolic pathway based on the KEGG database among 41 *Flagellimonas* strains, including the two novel isolates and 39 type strains (Fig. S4). Most of the *Flagellimonas* strains were similar in amino acid utilization, ethanol fermentation, fatty acid degradation, fermentation, oxidative phosphorylation, and arsenic cycling. However, there are also significant differences in genes related to nitrogen cycling and complex carbon degradation, reflecting distinct metabolic strategies. In nitrogen cycling, most of the strains, including strain C4^T^, were not able to reduce nitrate and nitrite, while some strains, such as strain GZD32^T^, contained genes for the reduction of nitrite to ammonia. As for complex carbon degradation, most of the strains exhibit a broad capacity for degrading various carbon sources, including cellulose, hemicellulose, chitin, and other oligosaccharides, but the gene counts are greatly different. For example, strain C4^T^ was devoid of hemicellulose-degrading genes, while strain GZD32^T^ possessed seven related genes. This diverse enzymatic ability enables them to break down a wide range of complex carbohydrates, contributing significantly to the recycling of organic carbon in the ecosystem.

**Fig 2:**
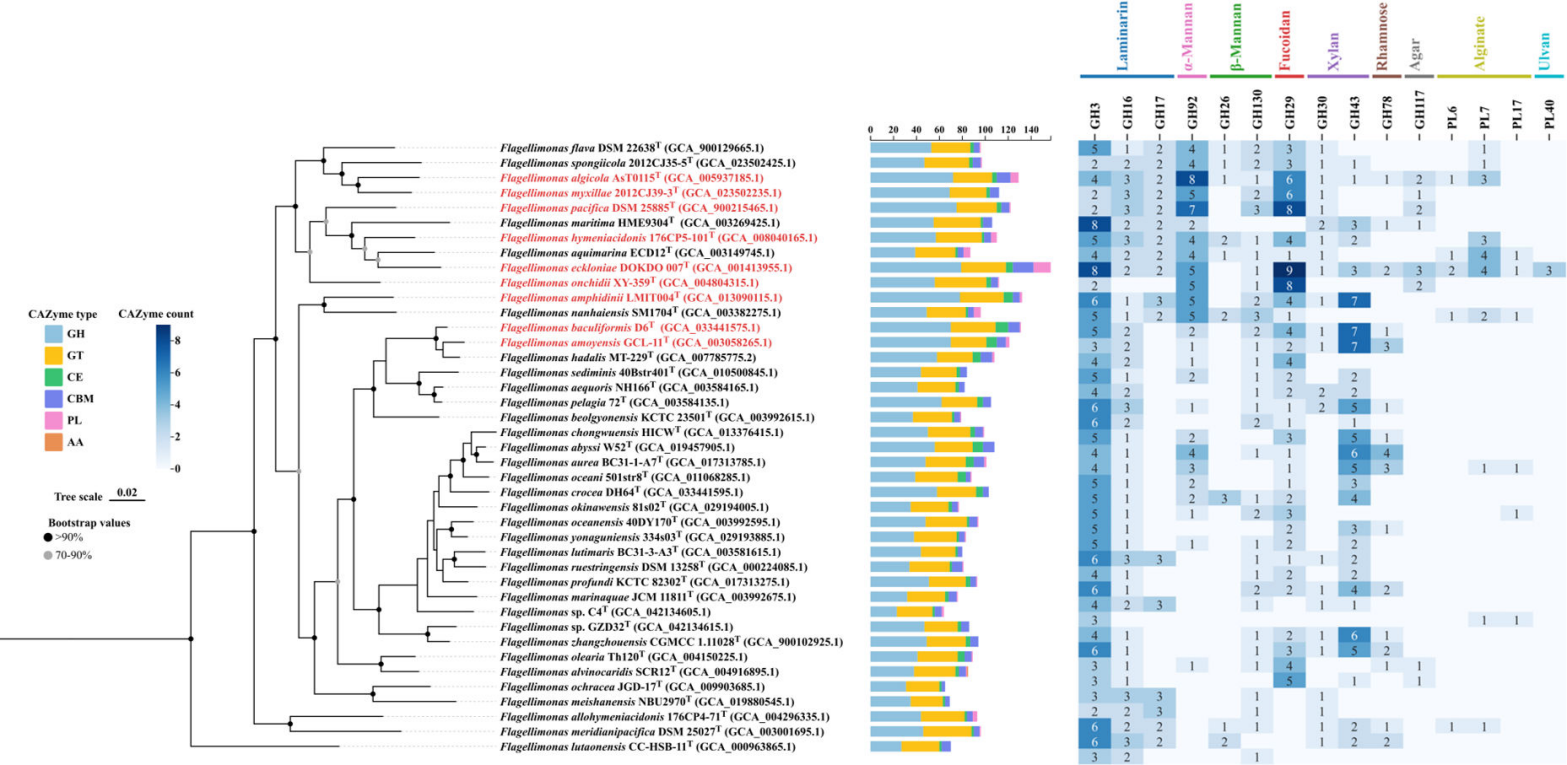
The phylogenetic tree based on genome sequences using GTDB-tk shows the relationships between strains C4^T^, GZD32^T^, and related taxa in genus *Flagellimonas* and the differences in their profile of CAZymes. Bar, 0.02 substitutions per amino acid position. *Zeaxanthinibacter enoshimensis* DSM 18435^T^ (GCA_004362865.1) was used as an outgroup (not shown). Strains in red indicate that the number of CAZymes annotated in the strain exceeds 110. The quantities and categories sum of CAZymes (middle panel) include GH (blue), GT (yellow), CE (green), CBM (purple), PL (pink), and AA (brown). The numbers and the shade (right panel) mean the number counts and relative abundance of selected CAZyme families.

To further compare the differences of *Flagellimonas* strains in complex carbon degradation, we performed the annotation of CAZyme, peptidase, and sulfatase and calculated their gene densities. The gene density of CAZyme, sulfatase, and peptidase of *Flagellimonas* strains ranged from 17.3 to 38.3 per Mb, 43.6–76.0 per Mb, and 55.2–75.4 per Mb, respectively, and the P/C ratio ranges from 1.66 to 3.31 (Table S3). To find out the distribution pattern of CAZymes in genus *Flagellimonas*, we analyzed the CAZymes according to the categories (Fig. 2). The number of CAZymes in this genus ranged from 64 to 157, and 9 strains contained more than 110 CAZymes. The glycoside hydrolase (GH) was widely distributed in this genus; by contrast, polysaccharide lyase (PL) was not annotated in 13 strains. As for strains, *F. eckloniae* DOKDO 007^T^ and strain C4^T^ had the maximum and minimum number of CAZymes, respectively.

Considering the specific substrates of CAZymes, we also predicted the potential substrates (mainly algal polysaccharides) of *Flagellimonas* strains (Fig. 2; Table S4). Statistical analysis revealed that the majority of *Flagellimonas* strains (> 70%) harbored GH3, GH16, GH130, GH29, and GH43 families, which are associated with the degradation of laminarin, β-mannan, fucoidan, and xylan. A small proportion of strains (>40%) contained GH17, GH30, GH92, and GH78 families, which are involved in the degradation of laminarin, xylan, α-mannan, and rhamnose, respectively. Notably, the GH92 family was present in relatively high abundance and exhibited a distinct clustering pattern among strains. By contrast, the GH117 family (which specifically recognizes and hydrolyzes β−1,4-glucosidic bonds in agar), the GH26 family (β-mannanase), and the PL6, PL7, and PL17 families (involved in alginate degradation) were detected in only a small fraction of strains (<20%). Furthermore, the PL40 family, which is associated with ulva polysaccharide degradation, was only identified in one strain.

Based on the distribution and abundance of CAZymes in the genus *Flagellimonas*, four typical and representative algal polysaccharide utilization loci (PULs) associated with alginate, fucoidan, ulvan, and agar were selected for prediction (Table S5). The results revealed significant variations in the ability of different *Flagellimonas* strains to degrade various types of algal polysaccharides. Specifically, 11 strains were predicted to harbor alginate-related PULs, while another 11 strains possessed fucoidan-related PULs. By contrast, only one strain exhibited ulvan-related PUL, and two strains were predicted to have agar-related PULs. Overall, *Flagellimonas* demonstrates a strong metabolic potential for brown algal polysaccharide degradation, highlighting its ecological adaptation to brown algae-rich environments. Among the primary polysaccharides in brown algae, for example, laminarin, fucoidan, and alginate, alginate has a relatively simple structure (67, 68). We decided to choose alginate-related PULs for further evolutionary analyses.

### Prediction of the alginate utilization locus in the genus *Flagellimonas*

**Fig 3:**
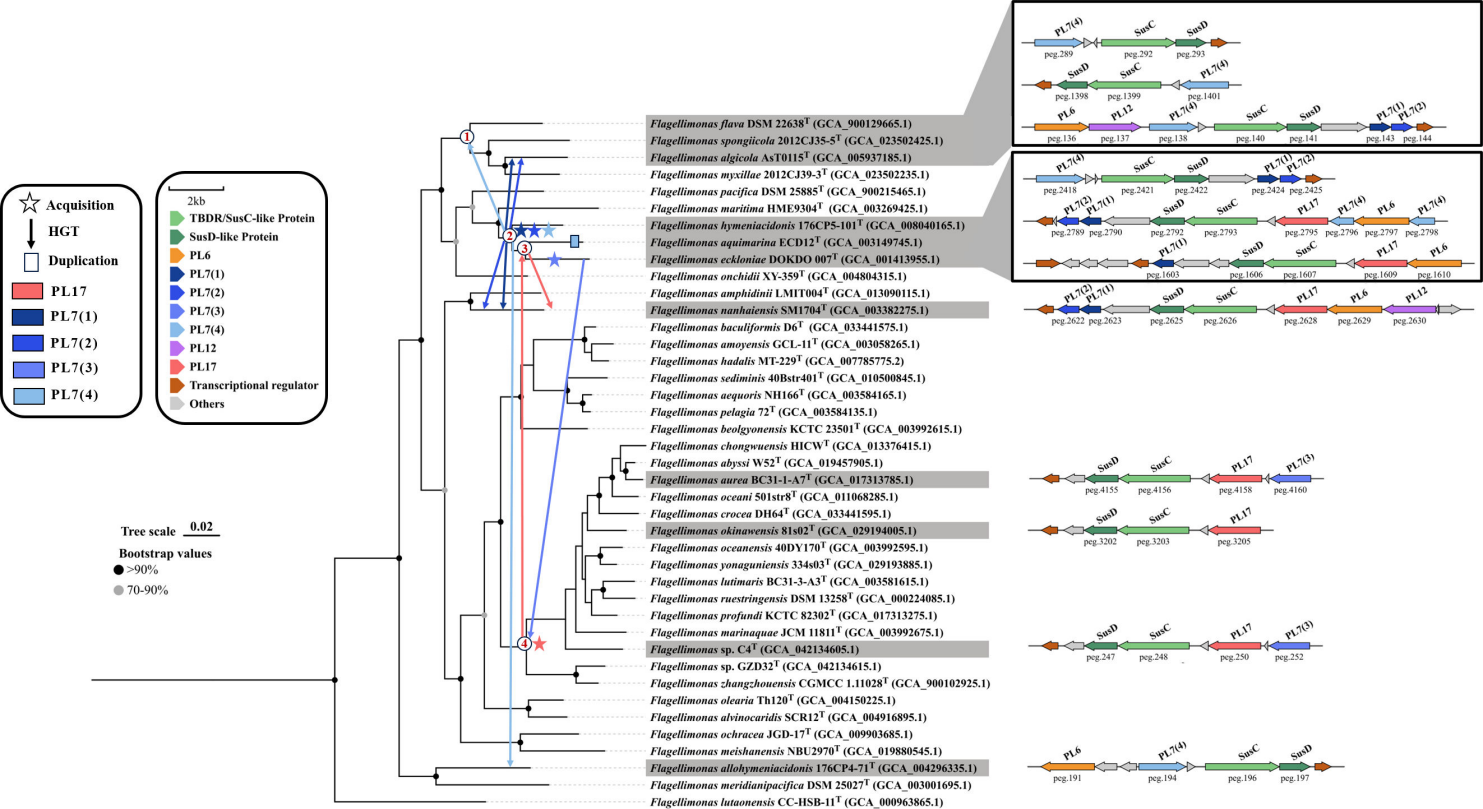
The profiles of alginate utilization loci (AUL) in *Flagellimonas* strains and the predicted evolutionary events of key genes in AULs using the phylogenetic reconciliation method. Bar, 0.02 substitutions per nucleotide position. Nodes with bootstrap values greater than 90 are displayed in black, while those with values between 70 and 90 are shown in gray. The strains in the gray background indicate that these strains possess alginate utilization loci. The numbers on the nodes represent identifiers, such as node ①; the arrows indicate the direction of horizontal gene transfer (HGT); the pentagrams and squares on the branches represent evolutionary events that occurred in the respective species; the panel on the right shows the structure of AUL.

According to the existence of PLs and SusC/D pairs, a total of 11 alginate utilization loci (AULs) were predicted in the *Flagellimonas* strains (Fig. 3). Each AUL contained genes encoding a pair of SusC/D proteins and at least one polysaccharide lyase (PL7 or PL17). The SusC/D pair was primarily responsible for the recognition and transport of alginate, while PL7 or PL17 played a key catalytic role in degrading alginate. In the phylogenetic tree based on the SusC/D gene sequences, all SusC or SusD genes in 11 AULs were clustered within a clade (Fig. S5), which confirmed the prediction of AULs due to the substrate specificity of SusC/D pairs (16). The 11 AULs were separately distributed in 11 different strains, in which *F. hymeniacidonis* 176CP5-101^T^, *F. aquimarina* ECD12^T^, and *F. eckloniae* DOKDO 007^T^ were located in one branch and shared a similar complicated structure of AULs, and *F. aurea* BC31-1-A7^T^, *F. okinawensis* 81s02,^T^ and strain C4^T^ were located in one large branch and contained similar AULs with a simple constitution. By contrast, *F. flava* DSM 22638^T^, *F. spongiicola* 2012CJ35-5^T^, and *F. algicola* AsT0115^T^ were closely related to each other, and the simple AUL that existed both in *F. flava* DSM 22638^T^ and *F. spongiicola* 2012CJ35-5^T^ was quite distinct from the AUL of *F. algicola* AsT0115^T^. In addition, even though *F. nanhaiensis* SM1704^T^ is phylogenetically far from AUL-containing strains, the AUL in *F. nanhaiensis* SM1704^T^ was complicated and shared similar structures of AUL in *F. algicola* AsT0115^T^, *F. hymeniacidonis* 176CP5-101^T^, *F. aquimarina* ECD12^T^, and *F. eckloniae* DOKDO 007^T^. These results indicated that the AUL distribution in *Flagellimonas* strains may experience several comprehensive evolution events.

To explore the evolution events of AULs in this genus, the evolutionary analysis of AULs and their key CAZymes was performed. The results revealed that, except for PL6, which does not exhibit a clear pattern of vertical inheritance, PL17 and PL7 displayed strong evidence for vertical inheritance (Fig. 3; Fig. S6). Nodes ①–④ in the above phylogenetic trees represented the most recent common ancestors of four clades within the genus *Flagellimonas*. The PL17 in *Flagellimonas* strains was first acquired by node ④ and subsequently transmitted to its descendants through vertical inheritance, resulting in the presence of PL17 in *F. okinawensis* 81s02^T^, strain C4^T^, and *F. aurea* BC31-1-A7^T^, whereas it was lost in other descendants of node ④. Then, PL17 was horizontally transferred from node ④ to node ③ and subsequently retained through vertical inheritance in the descendants of node ③, that is, *F. aquimarina* ECD12^T^ and *F. eckloniae* DOKDO 007^T^, and transferred to *F. nanhaiensis* SM1704^T^ through horizontal gene transfer (HGT).

While the PL7 exhibited a more complex pattern during evolution, primarily driven by vertical inheritance, followed by gene mutations and loss events. First, based on sequence similarities and phylogenetic relationships of PL7 proteins, PL7 was classified into four categories, that is, PL7(1), PL7(2), PL7(3), and PL7(4) (Fig. S6; Table S6). PL7(1), PL7(2), and PL7(4) were all initially originated from node ②, and PL7(1) and PL7(4) were likely acquired through horizontal gene transfer (HGT) from outside, while PL7(2) may have arisen from the duplication of PL7(1). The descendants of node ②, that is, *F. hymeniacidonis* 176CP5-101^T^, *F. aquimarina* ECD12^T^, and *F. eckloniae* DOKDO 007^T^, each contained one PL7(1), PL7(2), and PL7(4), while PL7(4) in *F. eckloniae* DOKDO 007^T^ (peg.3640) was separated from AUL but still existed in the genome. Besides the vertical inheritance, HGT from node ② to other strains also transferred the different categories of PL7. For instance, HGT from node ② to *F. algicola* AsT0115^T^ and *F. nanhaiensis* SM1704^T^, resulting in the acquisition of PL7(1) and PL7(2) in these strains, HGT from node ② to *F. allohymeniacidonis* 176CP4-71^T^ with PL7(4). Interestingly, HGT from node ② and node ①, then followed by vertical inheritance, allowed the descendants of node ①, that is, *F. flava* DSM 22638^T^, *F. algicola* AsT0115^T^, and *F. spongiicola* 2012CJ35-5^T^, to contain PL7(4). However, another descendant of node ①, which was absent from AUL, that is, *F. myxillae* 2012CJ39-3^T^, may have undergone gene loss. Gene duplication and mutation also play a crucial role in the evolutionary process. For example, the PL7(4) in *F. aquimarina* ECD12^T^ (*F. aquimarina*|peg.2798) was acquired through vertical inheritance as described above; it subsequently underwent gene duplication and resulted in the appearance of PL7(4) (*F. aquimarina*|peg.2796). Moreover, in *F. eckloniae* DOKDO 007^T^, PL7(2) may undergo a mutation, resulting in the formation of a new PL7 category, that is, PL7(3), namely *F. eckloniae*|peg.729. Subsequently, PL7(3) combined with a new CBM32 domain was horizontally transferred to node ④, which was further transmitted to its descendants (strains C4^T^ and *F. aurea* BC31-1-A7^T^) through vertical inheritance. Gene loss also occurred widely in *Flagellimonas* strains, such as the gene loss of PL7(3) in the descendants of node ④ and PL7(4) in *F. myxillae* 2012CJ39-3^T^.

Overall, we found two independent acquisitions of AUL within the genus *Flagellimonas* (Fig. S7). The first one happened in the ancestral node ②. It acquired two major function genes for PL7(1) and PL7(4) through horizontal gene transfer from other genera. Another independent acquisition of AUL happened in the ancestral node ④ with the gene for PL17 from outside. The AULs in ancestral nodes ① and ③ were mainly horizontally transferred or inherited from node ②, respectively, but the PL17 in ancestral node ③ was horizontally transferred from node ④. Then, the descendants of these nodes inherited the AULs with additional gene events such as gene mutations, duplication, or loss (Fig. 3).

Horizontal gene transfer (HGT) is considered the main reason for the gene diversity in *Flavobacteriaceae* genomes (69), such as the deep-sea *Flavobacteriaceae* strain *Mesoflavibacter profundi* MTRN7 may acquire its differential PUL by HGT from coastal or terrestrial *Bacteroidota* (70). A novel carrageenan metabolic pathway in *Flavobacterium algicola* widely exists in *Flavobacteriaceae* (71). However, the wide distribution of PL17 and PL7 in *Flagellimonas* strains primarily relies on vertical inheritance, although they have also undergone a certain degree of HGT. A similar report in *Myroides profundi* D25, whose initial adaptation to the marine environment relies primarily on vertically inherited genes rather than HGT events (72). During long-term evolution, *Flagellimonas* strains may rely more on stable genetic transmission to maintain the integrity of alginate degradation, such as the AUL of *F. nanhaiensis* SM1704^T^, which represents the earliest AUL structure, rather than depending on the frequent acquisition of exogenous genes.

### Functional assessment of AULs with different structures

**Fig 4:**
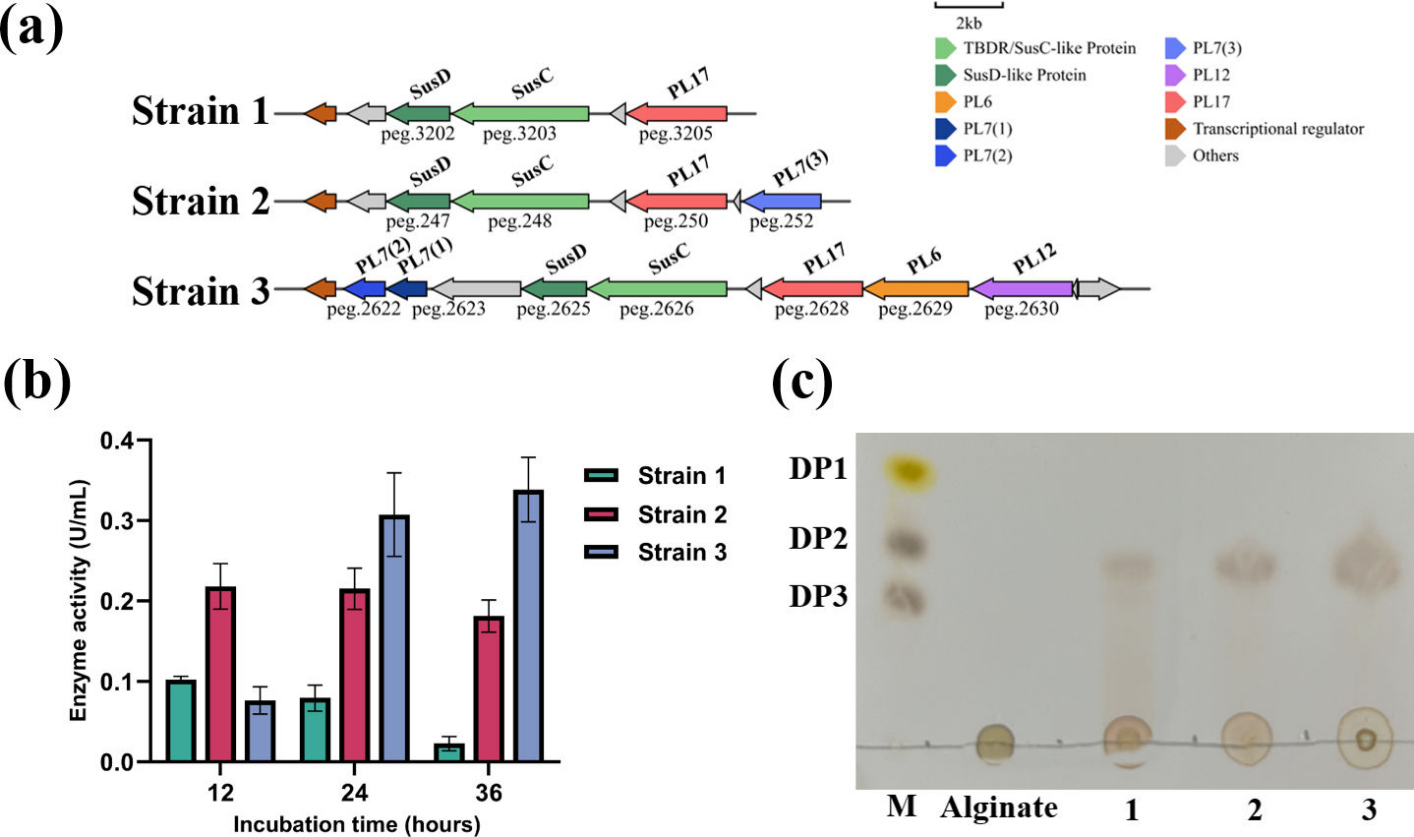
The enzymatic assessment of three strains containing representative alginate utilization loci (AUL) (**a**) Genetic structure of representative AULs; (**b**) Crude alginate lyase activity at different incubation times (12 h, 24 h, and 36 h) measured using the DNS method; (**c**) alginate degradation product analysis by TLC method. Strain 1: *Flagellimonas okinawensis* MCCC 1K08502^T^; Strain 2: Strain C4^T^; Strain 3: *Flagellimonas nanhaiensis* MCCC 1K03557^T^. M indicates mixed saccharide markers in different degrees of polymerization (DP) with rhamnose (DP1), sucrose (DP2), and melitriose (DP3); alginate indicates a negative control without inoculation.

We found that the AUL structures in *Flagellimonas* strains also show a trend from complex to simplified, especially in the descendants of nodes ① and ④ (Fig. 3). Based on the evolutionary analysis of the AULs, three strains were selected as the representing strains during different stages in the evolution to provide a comprehensive understanding of how variations in AUL structure correlate with the capabilities in alginate degradation. Among them, AUL in *F. nanhaiensis* SM1704^T^ was complicated in its structure, and it may represent the earliest AUL structure in the genus. The AUL in strain C4^T^, with the deletion of PL6, PL12, and one PL7 compared to *F. nanhaiensis* SM1704^T^, exhibited a stage of functional loss in the AUL evolutionary. The AUL in *F. okinawensis* 81s02^T^ had a simpler structure than strain C4^T^, with the further loss of one PL7 (Fig. 4a).

The crude alginate lyase of *F. nanhaiensis* SM1704^T^, strain C4,^T^ and *F. okinawensis* 81s02^T^ was assessed. The results showed that under induction by alginate, the highest enzyme activities of the three strains were 0.338 ± 0.040 U, 0.218 ± 0.028 U, and 0.102 ± 0.004 U, respectively (Fig. 4b). The statistical analysis showed that *F. nanhaiensis* SM1704^T^ was significantly higher than strain C4^T^ (*P* < 0.05), and strain C4^T^ was also significantly higher than *F. okinawensis* 81s02^T^ (*P* < 0.05) in the highest enzyme activity of crude alginate lyase. It was further confirmed by the TLC analysis of degradation products. The crude alginate lyase from three strains also showed the rank of *F. nanhaiensis* SM1704^T^ > strain C4^T^ >*F. okinawensis* 81s02^T^ (Fig. 4c). In addition, the growth curves in medium using alginate as the sole carbon source showed that *F. nanhaiensis* SM1704^T^ and strain C4^T^ could use alginate to support their stable and rapid growth, and the descent in the growth curve of *F. okinawensis* 81s02^T^ after 12 hours also revealed its weak ability in alginate degradation (Fig. S8). As a result, the multiple PLs in AUL of *F. nanhaiensis* SM1704^T^ work synergistically to efficiently degrade alginate; the sole PL17 in AUL of *F. okinawensis* 81s02^T^ could not degrade alginate efficiently; the gradual loss of PL7 in *Flagellimonas* strains resulted in the reduction of alginate degradation ability from *F. nanhaiensis* SM1704^T^ to strain C4^T^ and then *F. okinawensis* 81s02^T^.

Strains with the trend from high-efficiency degradation to low-efficiency degradation may decrease their need for alginate as a carbon source (73) and closely relate to the availability of alginate in the environment and the changes in bacterial survival strategies (74). The loss of certain alginate lyases in bacteria could also be an adaptive evolutionary strategy to transfer their roles from external hydrolyzers to selfish bacteria, thereby reducing metabolic costs (21), since the PL7 genes are mainly responsible for the initial depolymerization of alginate (74). We suppose that most *Flagellimonas* strains, especially descendants of node ④, may gradually lose the alginate degradation ability for niche adaptation.

### Conclusions

In this study, strains C4^T^ and GZD32^T^ were polyphasic characterized and proposed as type strains of two novel species, *Flagellimonas alginolytica* sp. nov. and *Flagellimonas cixiensis* sp. nov., respectively. The comparative genomic analysis revealed that *Flagellimonas* strains were diverse in their ability of complex carbon degradation and exhibited a preference for polysaccharides derived from brown algae. Further evolutionary analysis of function genes in AULs indicated that *Flagellimonas* strains acquired AULs through HGT in the early stage, but the wide distribution of PL17 and PL7 in this genus primarily relies on vertical inheritance. The AUL structures in *Flagellimonas* strains showed the trend of simplification, which was testified to be correlated with the reduction in alginate-degrading ability, indicating that the gradual loss of AUL genes was a strategy for niche adaptation of *Flagellimonas* strains.

### Description of *Flagellimonas alginolytica* sp. nov

*Flagellimonas alginolytica* (al.gi.no.ly.ti.ca. N.L. neut. n. *acidum alginicum*, alginic acid; N.L. fem. adj. *lytica*, dissolving; N.L. fem. adj. *alginolytica*, alginic acid-dissolving.)

Cells are Gram-stain-negative, facultative anaerobic, motile with flagella. Strain is oxidase- and catalase-positive. Cells are rod-shaped, measuring 2.3–3.2 µm in length and 0.4–0.8 µm in width. Colonies are yellow, round, opaque, with smooth borders and a convex shape. Growth is observed at temperatures 15°C–40°C (optimum, 37°C), pH 5.5–10 (optimum, pH 7.0), and in the presence of 0–10.0 % (wt/vol) NaCl (optimum, 2.0%). Hydrolyze starch, Tween 40, 60, 80, and tyrosine, but not Tween 20, cellulose, or casein. In the API 20NE test, positive for nitrate reduction to nitrite, esculin hydrolysis, gelatin hydrolysis, and β-galactosidase activity. In the API ZYM test, the following enzymes tested positive: alkaline phosphatase, esterase (C4), esterase lipase (C8), leucine arylamidase, valine arylamidase, trypsin, acid phosphatase, naphthol-AS-BI-phosphohydrolase, α-galactosidase, β-galactosidase, α-glucosidase, β-glucosidase, N-acetyl-β-glucosaminidase, and α-mannosidase. Produce acid from glycerol, L-arabinose, D-ribose, D-xylose, D-galactose, D-glucose, D-fructose, D-mannose, D-mannitol, methyl-αD-mannopyranoside, methyl-αD-glucopyranoside, N-acetyl-glucosamine, amygdalin, arbutin, esculin ferric citrate, D-cellobiose, D-maltose, D-lactose, D-melibiose, D-saccharose, D-trehalose, inulin, D-melezitose, D-raffinose, starch, glycogen, gentiobiose, D-turanose, and D**-**tagatose. D-maltose, tyrosine, and alginate can be used as sole carbon substrates. The major fatty acids (>10%) include iso-C_15:0_, iso-C_17:0_ 3-OH, and iso-C_15:1_ G. The major respiratory quinone is MK-6. The main polar lipids include phosphatidylethanolamine, aminophosphoglycolipid, and three unidentified lipids. The G + C content of the strain is 41.3%.

The type strain C4^T^ (=MCCC M28979^T^=KCTC 92864^T^) was isolated from a seawater mixture sample collected from the Yellow Sea (35°40′ N, 121°00′ E). The GenBank accession numbers for the 16S rRNA gene and genome sequences of strain C4^T^ are PP762181 and CP169638.1, respectively.

### Description of *Flagellimonas cixiensis* sp. nov

*Flagellimonas cixiensis* (ci.xi.en’sis. N.L. masc./fem. adj. *cixiensis*, pertaining to Cixi, PR China, where the strain was isolated.)

Cells are Gram-stain-negative, facultative anaerobic, non-motile, oxidase-negative, and catalase-positive. Cells are long, rod-shaped, with a length of 1.7–2.6 μm and a width of 0.2–0.3 μm. Colonies are orange, round, opaque, with smooth borders and a convex shape. Growth is observed at temperatures 18°C–40°C (optimum, 28°C), pH 5.5–8.5 (optimum, pH 7.0), and in the presence of 0.5%–8.0% (wt/vol) NaCl (optimum, 1.5%). Hydrolyze Tween 20, 40, 60, 80, and tyrosine, but not starch, cellulose, or casein. In the API 20NE test, positive for nitrate reduction to nitrogen, esculin hydrolysis, gelatin hydrolysis, and β-galactosidase activity. In the API ZYM test, the following enzymes tested positive: alkaline phosphatase, esterase (C4), esterase lipase (C8), lipase (C14), leucine arylamidase, valine arylamidase, trypsin, acid phosphatase, naphthol-AS-BI-phosphohydrolase, α-galactosidase, β-galactosidase, α-glucosidase, β-glucosidase, N-acetyl-β-glucosaminidase, and α-mannosidase. Produce acid from L-arabinose, D-xylose, D-galactose, D-glucose, D-fructose, D-mannose, methyl- αD-mannopyranoside, methyl-αD-glucopyranoside, N-acetyl-glucosamine, amygdalin, arbutin**,** esculin ferric citrate, D-cellobiose, D-maltose, D-lactose, D-melibiose, D-saccharose, D-trehalose, D-melezitose, D-raffinose, starch, glycogen, gentiobiose, and D-turanose. L-rhamnose monohydrate and tyrosine can be used as sole carbon sources. The major fatty acids (>10%) are iso-C_15:0_, iso-C_17:0_ 3-OH, and Summed Feature 8 (C_18:1_ *ω*6*c* and/or C_18:1_ ω*7c*). The major respiratory quinone is MK-6. The major polar lipids consist of phosphatidylethanolamine, aminophosphoglycolipid, two unidentified lipids (L1 and L2), and one unidentified glycolipid (GL). The G + C content of the strain is 41.3%.

The type strain GZD32^T^ (=MCCC 1K09106^T^=KCTC 102298^T^) was isolated from tidal flat sediment collected in Cixi, Zhejiang Province, China (30°10′ N, 121°14′ E). The GenBank accession numbers for the 16S rRNA gene and genome sequences of strain GZD32^T^ are PP762183 and CP169639.1, respectively.

## Data Availability

The complete genome sequences of strain C4^T^ and GZD32^T^ have been deposited in GenBank under the accession numbers CP169638.1 and CP169639.1, respectively. The 16S rRNA gene sequences of strain C4^T^ and GZD32^T^ have been deposited in GenBank under the accession numbers PP762181 and PP762183, respectively.

## SUPPLEMENTAL MATERIAL

Supplemental material spectrum.00917-25-s0001.pdf Fig. S1 to S8; Tables S1 to S3.

Table S4 spectrum.00917-25-s0002.xlsx The information about annotated CAZymes and SusC/D proteins.

Table S5 spectrum.00917-25-s0003.xlsx Information about functional genes in predicted PULs.

Table S6 spectrum.00917-25-s0004.xls The dbCAN annotation results about PL7.

ASM does not own the copyrights to Supplemental Material that may be linked to, or accessed through, an article. The authors have granted ASM a non-exclusive, world-wide license to publish the Supplemental Material files. Please contact the corresponding author directly for reuse.
