# Supplementary material for: Characterization of two novel species of the genus Flagellimonas reveals the key role of vertical inheritance in the evolution of alginate utilization loci
Source: Microbiol Spectr. 2025 Jul 7;13(8):e00917-25. doi: 10.1128/spectrum.00917-25 (PMC12323373; doi:10.1128/spectrum.00917-25)
Supplement: Supplemental material — Fig. S1 to S8; Tables S1 to S3. [file spectrum.00917-25-s0001.pdf]

Supplementary materials

Characterization of two novel species of genus *Flagellimonas* reveals the key role of vertical inheritance in the evolution of alginate utilization loci

Juan Yu[1], Jia-Wei Gao[1,2], Ke Cao[1], Dong-Yan He[1], Lin Xu[1,2], Ge-Yi Fu[3] and Cong Sun[1,2*]

[1]College of Life Sciences and Medicine, Zhejiang Sci-Tech University, Hangzhou 310018, PR China

[2]Shaoxing Biomedical Research Institute of Zhejiang Sci-Tech University Co., Ltd, Zhejiang Engineering Research Center for the Development Technology of Medicinal and Edible Homologous Health Food, Shaoxing 312075, PR China

[3]Key Laboratory of Marine Ecosystem Dynamics, Ministry of Natural Resources & Second Institute of Oceanography, Ministry of Natural Resources, Hangzhou 310012, PR China

Corresponding authors:

Cong Sun, michael_sc@sina.com

Fig. S1. The circular map of strains C4^{T} and GZD32^{T} showing general genomic features.

From the outermost ring to the innermost ring, the first and fourth rings represent coding sequences (CDS) on the forward and reverse DNA strands, with different colors denoting distinct COG (Cluster of Orthologous Groups) functional categories. The second and third rings depict CDS, tRNA, and rRNA on both the forward and reverse strands. The fifth ring displays the GC (Guanine-Cytosine) content, with outward extensions indicating regions where the GC content exceeds the genome-wide average GC content. The sixth ring represents GC-skew values, calculated using the formula (G − C)/ (G + C). The innermost ring serves as a size indicator for the genome.

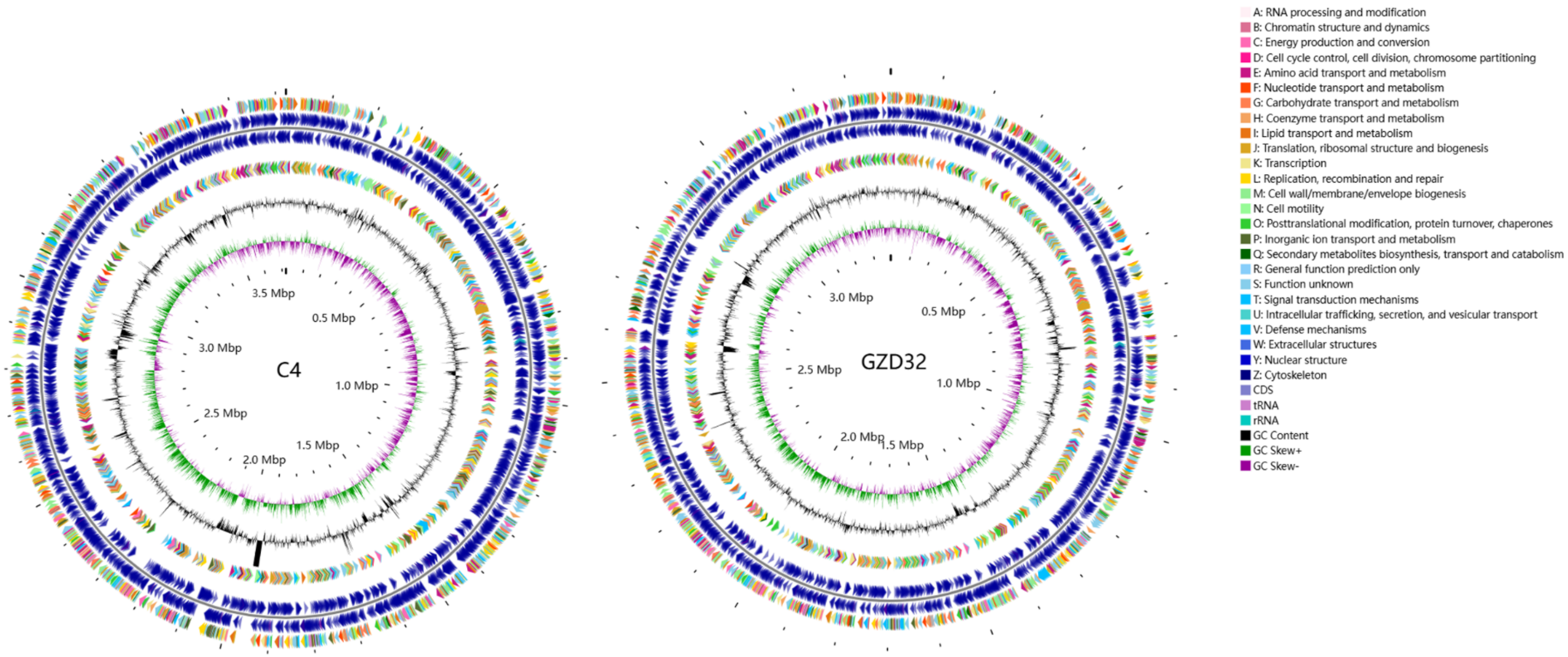

Fig. S2. Transmission electron microscope of cells of strains C4^T (a, b) and GZD32^T (c, d) grown on marine agar for 3 days at 30 °C.

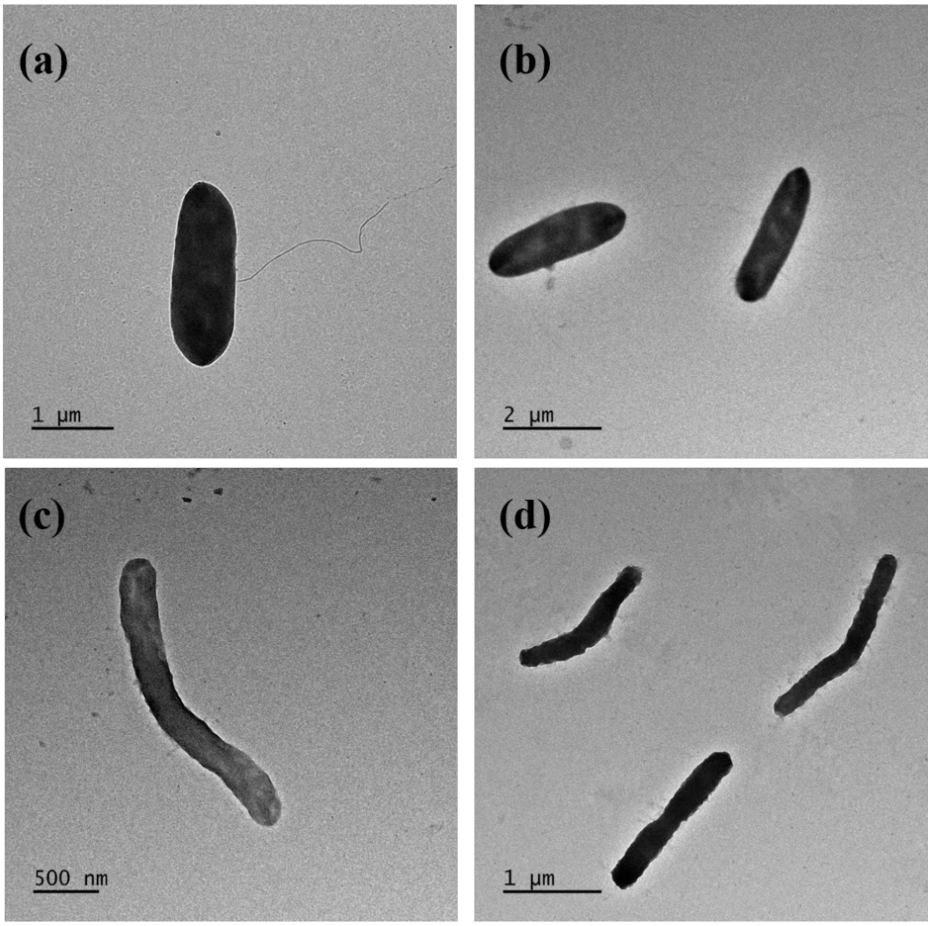

Fig. S3. Two-dimensional TLC plate images of total polar lipids of (A) strains C4^{T} and (B) GZD32^{T} with different spraying reagents (a) phosphomolybdic acid; (b) sulfuric acid; (c) ninhydrin; (d) molybdenum blue.

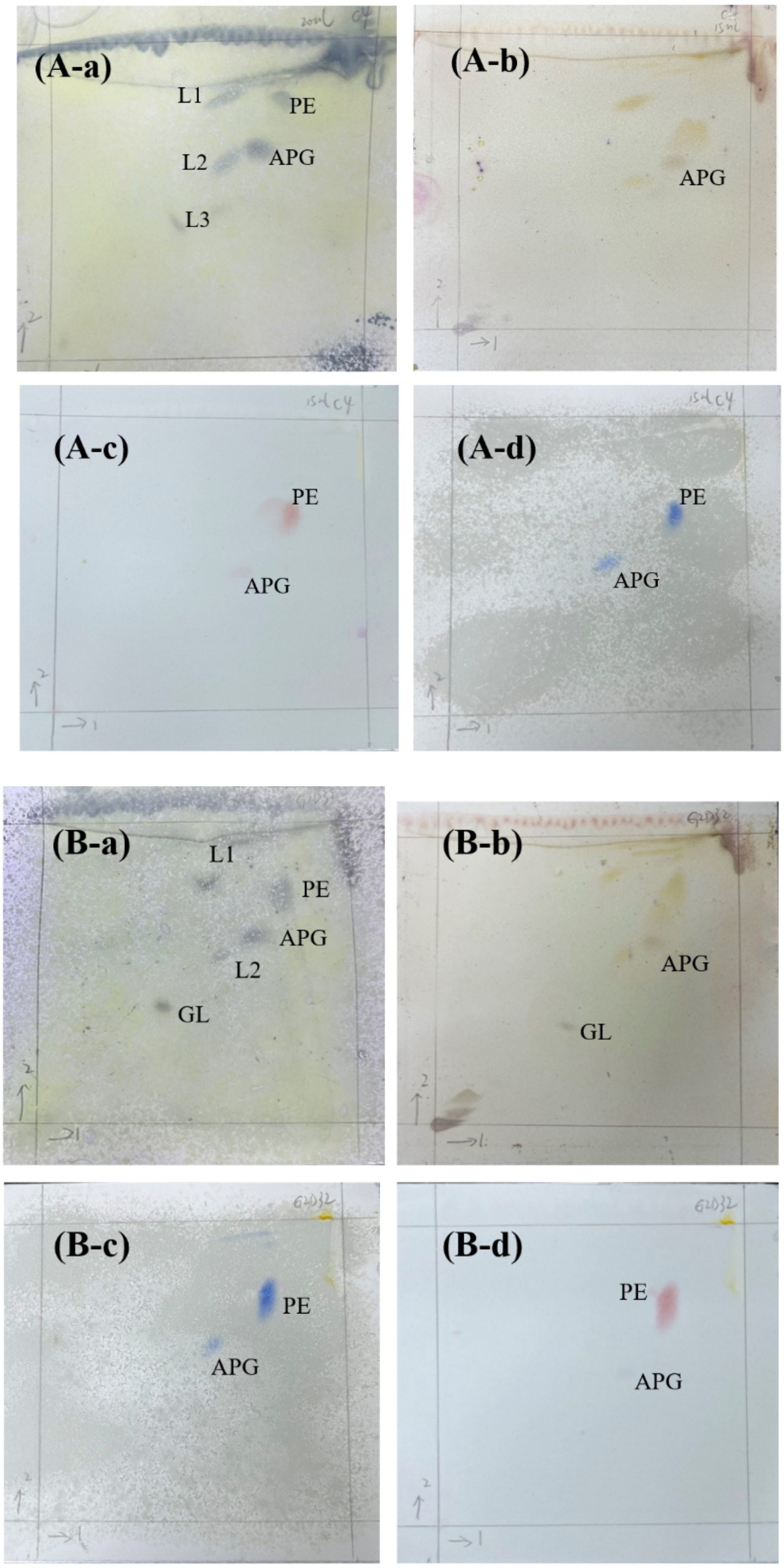

Fig. S4. Comparison of core metabolic genes of strains C4^{T}, GZD32^{T}and related *Flagellimonas* strains.

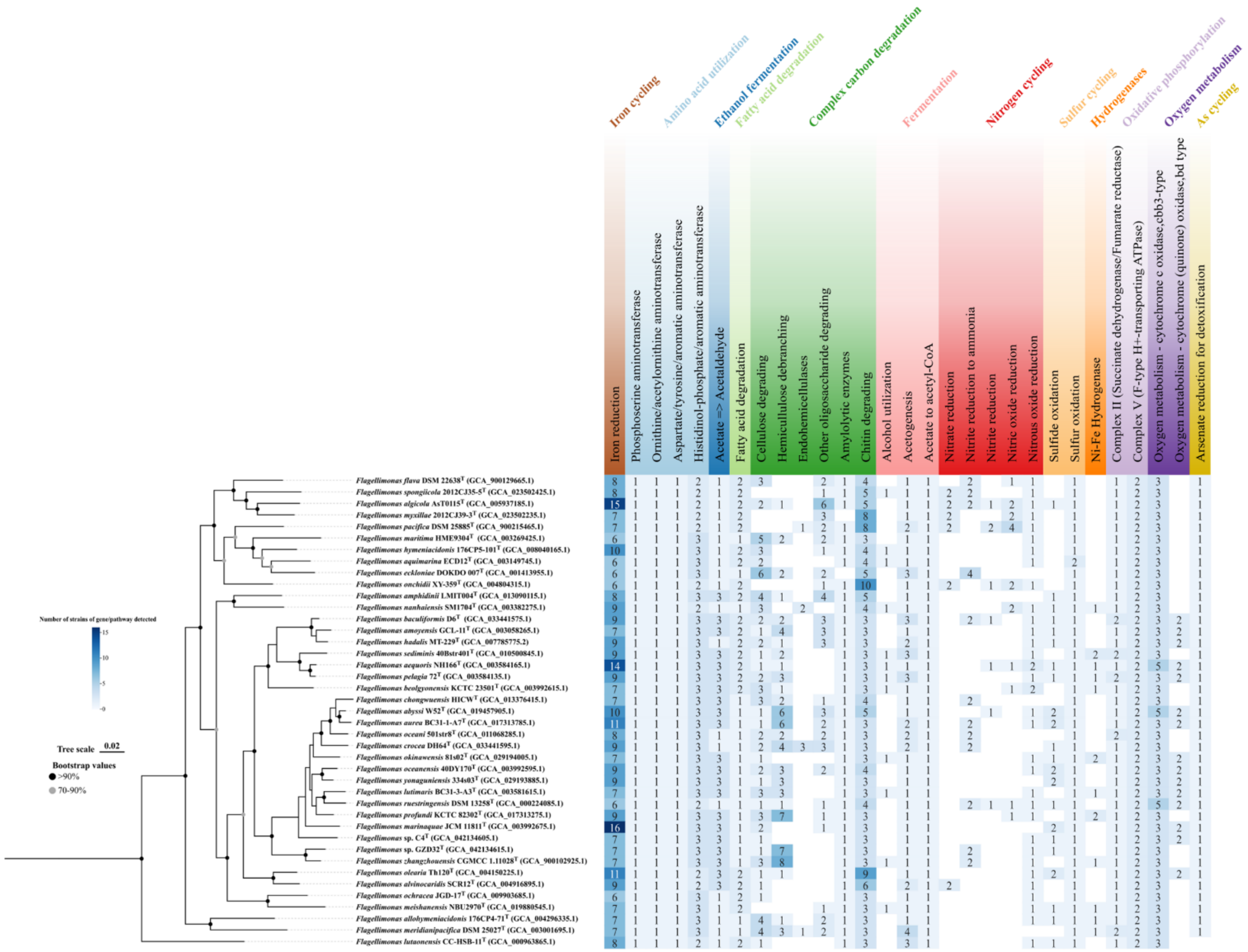

Fig. S5. The maximum-likelihood phylogenetic tree based on amino acids sequences of all SusC-like and SusD-like proteins in *Flagellimonas* strains showing the phylogenetic relationship of alginate related SusC-like and SusD-like proteins.

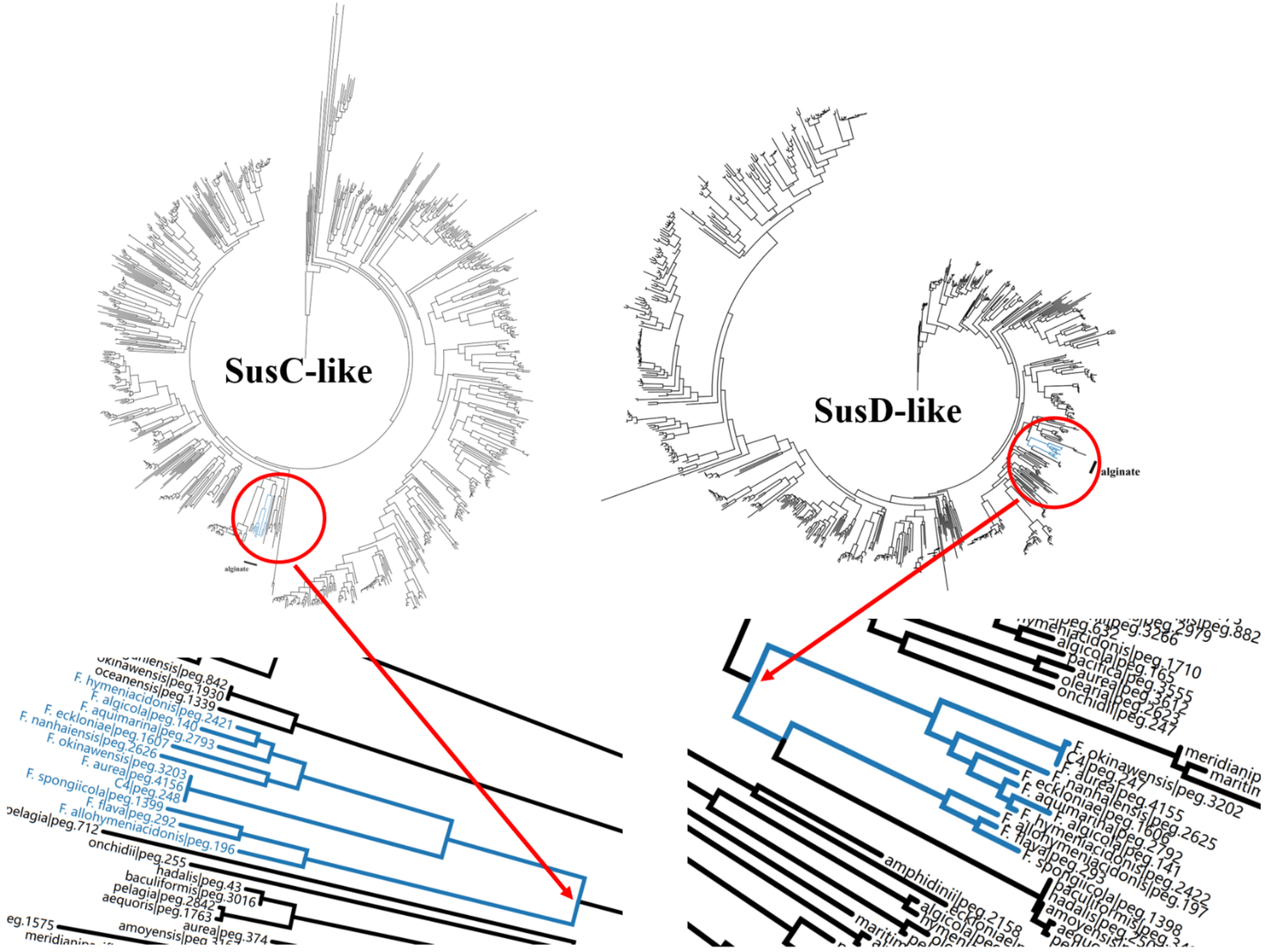

Fig. S6. The maximum-likelihood phylogenetic tree based on amino acid sequences of alginate lyases belonging to PL6, PL7 and PL17 families. The alginate lyase protein (F. nanhaiensis|peg.2630) belonging to PL12 family was used as the outgroup. Bar, 0.02 substitutions per amino acid position. The triangle mark indicates proteins are not located in the alginate utilization loci. The sequence identities between proteins were calculated by BLASTP, the identities between different families or PL7 categories were both below 50% (not shown).

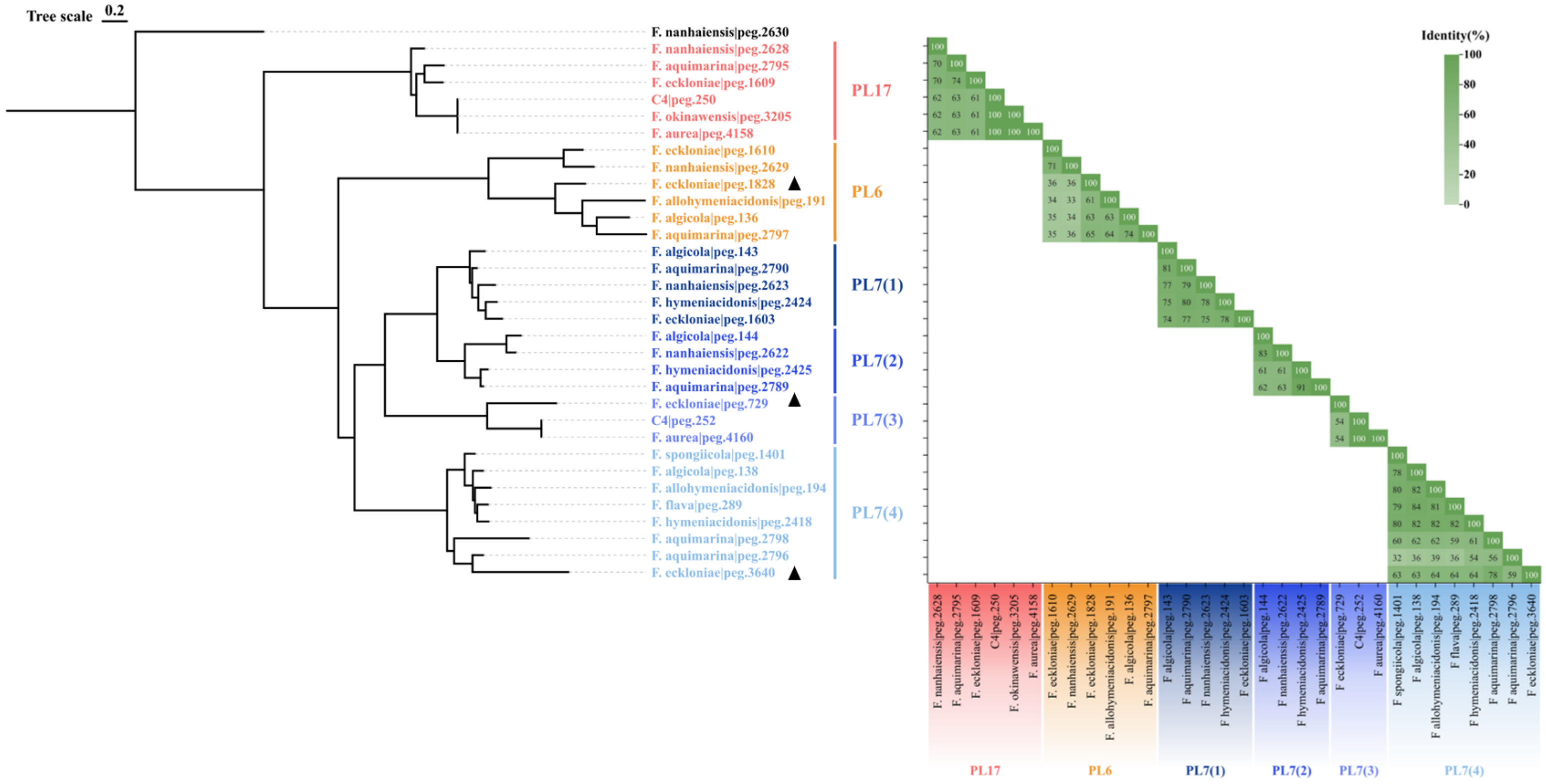

Fig. S7. Schematic representation of AULs-related gene modules between *Flagellimonas* species and ancestral nodes.

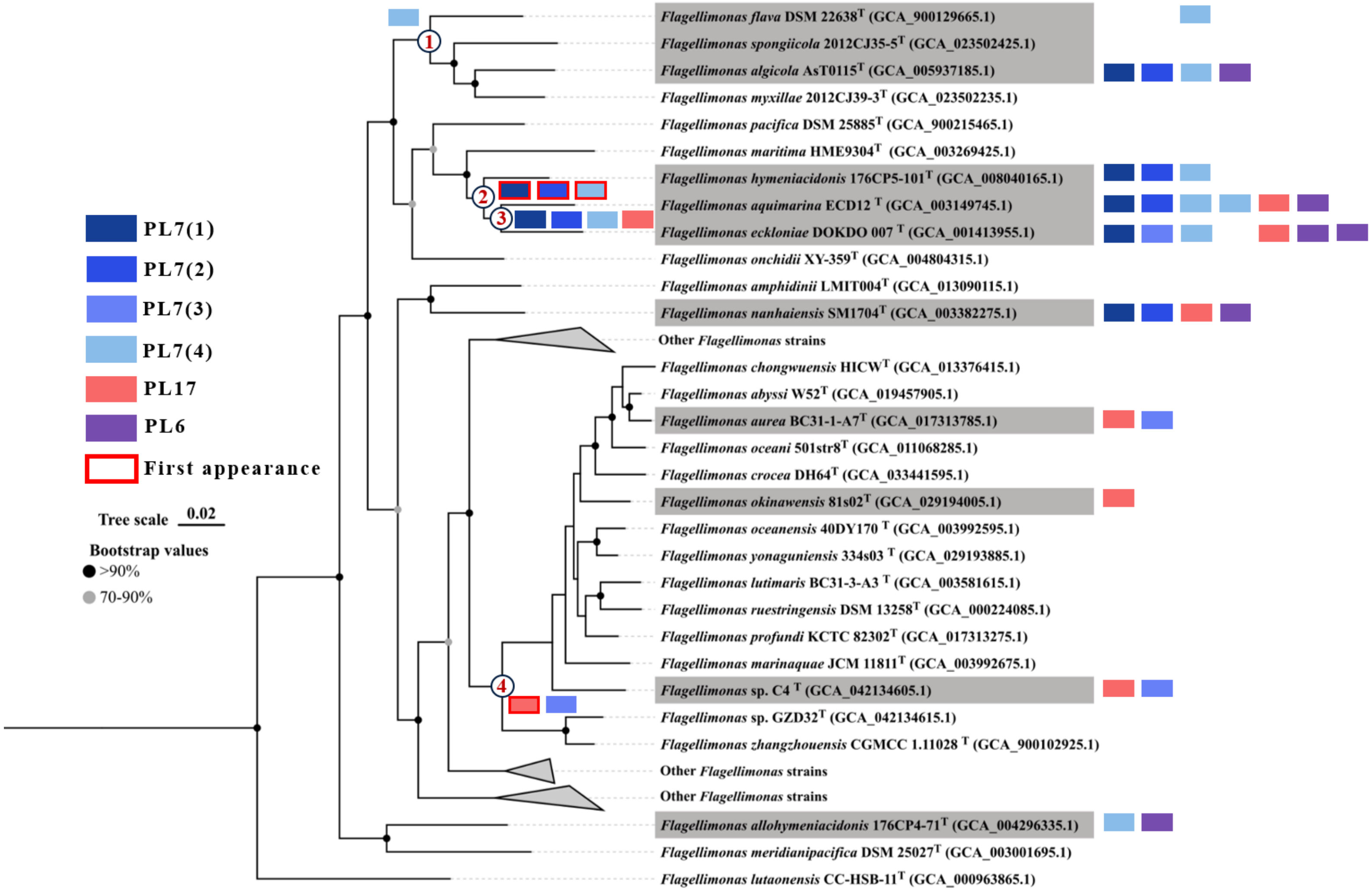

Fig. S8. The growth curves of the three strains cultured for 12 h, 24 h, and 36 h in a medium containing 1 g/L alginate as the sole carbon source.

Strain 1: *Flagellimonas okinawensis* MCCC 1K08502^{T}; Strain 2: Strain C4^{T}; Strain 3: *Flagellimonas nanhaiensis* MCCC 1K03557^{T}.

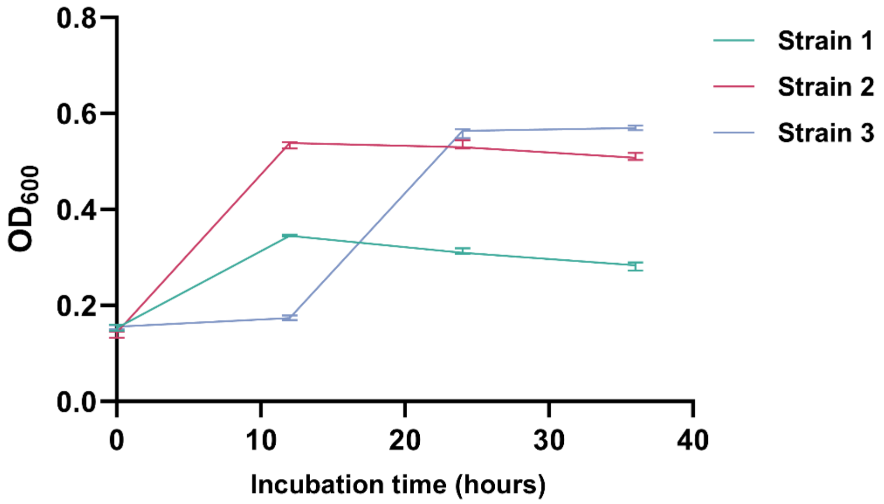

Table S1. The genomic relatedness indexes between strains C4^T, GZD32^T and type strains in genus *Flagellimonas*.

Species	Strain C4^T		Strain GZD32^T	
	ANI%	dDDH%	ANI%	dDDH%
Flagellimonas abyssi W52^T	80.6	21.4	79.6	20.0
Flagellimonas aequoris NH166^T	78.7	19.0	79.0	19.5
Flagellimonas algicola AsT0115^T	78.8	17.6	77.9	17.9
Flagellimonas alvinocaridis SCR12^T	78.5	18.6	78.7	19.1
Flagellimonas amoyensis GCL-11^T	78.5	18.7	78.7	18.9
Flagellimonas amphidinii LMIT004^T	77.5	17.9	78.1	18.3
Flagellimonas aurea BC31-1-A7^T	80.7	21.7	79.8	20.1
Flagellimonas baculiformis D6^T	79.1	19.4	78.5	18.9
Flagellimonas beolgyonensis KCTC 23501^T	78.5	18.7	78.9	19.3
Flagellimonas chongwuensis HICW^T	80.3	21.2	79.5	19.9
Flagellimonas crocea DH64^T	80.3	21.0	79.7	20.1
Flagellimonas hadalis MT-229^T	78.7	19.0	78.5	18.9
Flagellimonas lutimaris BC31-3-A3^T	80.7	21.9	79.8	20.5
Flagellimonas marinaquae JCM 11811^T	80.1	20.6	79.2	19.6
Flagellimonas meishanensis NBU2970^T	77.7	17.4	77.9	17.8
Flagellimonas myxillae 2012CJ39-3^T	77.6	17.4	77.8	17.7
Flagellimonas nanhaiensis SM1704^T	77.7	17.8	77.7	18.0
Flagellimonas oceanensis 40DY170^T	81.1	22.1	79.7	20.5
Flagellimonas oceani 501str8^T	80.8	22.1	79.6	20.0
Flagellimonas ochracea JGD-17^T	77.8	17.6	77.5	17.8
Flagellimonas okinawensis 81s02^T	80.9	21.7	80.1	20.2
Flagellimonas olearia Th120^T	78.5	18.6	78.9	19.1
Flagellimonas pacifica DSM 25885^T	77.6	17.8	77.9	18.3
Flagellimonas pelagia 72^T	78.8	19.2	79.0	19.4

Flagellimonas profundi KCTC 82302^T	81.4	22.5	79.6	20.1
Flagellimonas ruestringensis DSM 13258^T	80.6	22.0	79.7	20.5
Flagellimonas sediminis 40Bstr401^T	78.6	19.0	78.6	19.1
Flagellimonas yonaguniensis 334s03^T	81.2	22.4	79.9	20.3
Flagellimonas zhangzhouensis CGMCC 1.11028^T	79.3	19.6	85.9	29.9

Table S2. The basic genomic information of *Flagellimonas* strains used in this study.

Strains	Genome size (Mb)	G+C content (%)	Genome completeness (%)	Genome contamination (%)	NCBI GenBank
Flagellimonas abyssi W52^T	4.4	41.7	99.34	0.71	GCA_019457905.1
Flagellimonas aequoris NH166^T	4.2	43.4	99.50	1.97	GCA_003584165.1
Flagellimonas algicola AsT0115^T	4.5	41.8	99.68	0.81	GCA_005937185.1
Flagellimonas allohymeniacidonis 176CP4-71^T	3.6	41.7	99.34	0.00	GCA_004296335.1
Flagellimonas alvinocaridis SCR12^T	3.7	42.3	99.34	0.22	GCA_004916895.1
Flagellimonas amoyensis GCL-11^T	4.1	45.7	99.68	0.65	GCA_003058265.1
Flagellimonas amphidinii LMIT004^T	3.9	38.9	99.68	0.00	GCA_013090115.1
Flagellimonas aquimarina ECD12^T	3.6	37.6	99.35	0.49	GCA_003149745.1
Flagellimonas aurea BC31-1-A7^T	4.5	42.1	99.34	1.21	GCA_017313785.1
Flagellimonas baculiformis D6^T	4.3	45.5	99.03	0.00	GCA_033441575.1
Flagellimonas beolgyonensis KCTC 23501^T	3.8	43.6	99.67	2.07	GCA_003992615.1
Flagellimonas chongwuensis HICW^T	3.8	41.4	99.34	0.00	GCA_013376415.1
Flagellimonas crocea DH64^T	4.3	42.6	99.68	0.32	GCA_033441595.1
Flagellimonas eckloniae DOKDO 007^T	4.1	37.8	99.35	0.97	GCA_001413955.1
Flagellimonas flava DSM 22638^T	3.8	42.0	99.67	1.43	GCA_900129665.1
Flagellimonas hadalis MT-229^T	4.1	45.6	99.35	0.32	GCA_007785775.2
Flagellimonas hymeniacidonis 176CP5-101^T	3.9	37.8	99.35	0.65	GCA_008040165.1
Flagellimonas lutaonensis CC-HSB-11^T	3.3	45.0	99.35	0.32	GCA_000963865.1
Flagellimonas lutimaris BC31-3-A3^T	3.7	40.1	99.34	0.75	GCA_003581615.1
Flagellimonas marinaquae JCM 11811^T	3.4	43.4	99.35	0.39	GCA_003992675.1
Flagellimonas maritima HME9304^T	3.8	38.1	99.01	0.17	GCA_003269425.1
Flagellimonas meishanensis NBU2970^T	3.2	43.8	99.34	0.56	GCA_019880545.1
Flagellimonas meridianipacifica DSM 25027^T	4.4	39.7	99.32	0.21	GCA_003001695.1
Flagellimonas myxillae 2012CJ39-3^T	3.9	42.8	99.68	0.16	GCA_023502235.1
Flagellimonas nanhaiensis SM1704^T	4.0	40.7	99.67	0.76	GCA_003382275.1

Flagellimonas oceanensis 40DY170^T	4.3	42.4	99.34	0.50	GCA_003992595.1
Flagellimonas oceani 501str8^T	4.6	42.8	99.34	0.66	GCA_011068285.1
Flagellimonas ochracea JGD-17^T	3.6	41.1	99.34	0.59	GCA_009903685.1
Flagellimonas okinawensis 81s02^T	4.0	41.6	99.01	2.31	GCA_029194005.1
Flagellimonas olearia Th120^T	4.0	43.9	99.34	0.66	GCA_004150225.1
Flagellimonas onchidii XY-359^T	4.2	39.1	99.65	0.38	GCA_004804315.1
Flagellimonas pacifica DSM 25885^T	4.4	38.4	99.67	0.22	GCA_900215465.1
Flagellimonas pelagia 72^T	4.3	43.4	99.67	0.22	GCA_003584135.1
Flagellimonas profundi KCTC 82302^T	4.0	41.6	99.67	0.75	GCA_017313275.1
Flagellimonas ruestringensis DSM 13258^T	3.8	41.4	99.24	0.83	GCA_000224085.1
Flagellimonas sediminis 40Bstr401^T	4.2	42.9	99.34	2.48	GCA_010500845.1
Flagellimonas spongiicola 2012CJ35-5^T	3.7	39.6	99.01	1.16	GCA_023502425.1
Flagellimonas yonaguniensis 334s03^T	4.3	41.9	99.34	1.70	GCA_029193885.1
Flagellimonas zhangzhouensis CGMCC 1.11028^T	3.7	39.9	99.34	0.66	GCA_900102925.1
Flagellimonas sp. C4	3.7	41.3	99.34	0.73	GCA_042134605.1
Flagellimonas sp. GZD32	3.4	40.3	99.34	0.33	GCA_042134615.1

Table S3. The profile of peptidases and CAZymes of *Flagellimonas* strains used in this study.

Strains	CAZymes gene density (genes /Mb)	Peptidase gene density (genes/ Mb)	Peptidases/CAZYmes
Flagellimonas abyssi W52^T	24.5	62.7	2.56
Flagellimonas aequoris NH166^T	19.5	61.7	3.16
Flagellimonas algicola AsT0115^T	28.7	53.8	1.88
Flagellimonas allohymeniacidonis 176CP4-71^T	25.8	60.8	2.35
Flagellimonas alvinocaridis SCR12^T	23.0	58.1	2.53
Flagellimonas amoyensis GCL-11^T	29.5	58.0	1.97
Flagellimonas amphidinii LMIT004^T	33.8	56.2	1.66
Flagellimonas aquimarina ECD12^T	24.2	64.2	2.66
Flagellimonas aurea BC31-1-A7^T	22.4	63.1	2.81
Flagellimonas baculiformis D6^T	30.5	58.4	1.92
Flagellimonas beolgyonensis KCTC 23501^T	20.8	64.7	3.11
Flagellimonas chongwuensis HICW^T	26.1	61.1	2.34
Flagellimonas crocea DH64^T	24.0	60.2	2.51
Flagellimonas eckloniae DOKDO 007^T	38.3	75.4	1.97
Flagellimonas flava DSM 22638^T	25.3	60.8	2.41
Flagellimonas hadalis MT-229^T	26.3	56.6	2.15
Flagellimonas hymeniacidonis 176CP5-101^T	28.2	59.5	2.11
Flagellimonas lutaonensis CC-HSB-11^T	21.2	55.5	2.61
Flagellimonas lutimaris BC31-3-A3^T	21.6	68.9	3.19
Flagellimonas marinaquae JCM 11811^T	22.4	56.2	2.51
Flagellimonas maritima HME9304^T	27.9	56.1	2.01
Flagellimonas meishanensis NBU2970^T	21.6	61.9	2.87
Flagellimonas meridianipacifica DSM 25027^T	21.8	59.1	2.71
Flagellimonas myxillae 2012CJ39-3^T	28.7	57.9	2.02
Flagellimonas nanhaiensis SM1704^T	24.0	58.8	2.45
Flagellimonas oceanensis 40DY170^T	21.9	60.7	2.78
Flagellimonas oceani 501str8^T	19.1	57.0	2.98
Flagellimonas ochracea JGD-17^T	18.1	59.7	3.31

Flagellimonas okinawensis 81s02^T	19.3	62.3	3.23
Flagellimonas olearia Th120^T	22.3	59.5	2.67
Flagellimonas onchidii XY-359^T	26.7	56.7	2.13
Flagellimonas pacifica DSM 25885^T	27.7	55.2	1.99
Flagellimonas pelagia 72^T	24.4	63.5	2.60
Flagellimonas profundi KCTC 82302^T	23.3	60.3	2.59
Flagellimonas ruestringensis DSM 13258^T	21.3	65.3	3.06
Flagellimonas sediminis 40Bstr401^T	20.0	61.9	3.10
Flagellimonas spongiicola 2012CJ35-5^T	26.2	64.9	2.47
Flagellimonas yonaguniensis 334s03^T	19.3	61.4	3.18
Flagellimonas zhangzhouensis CGMCC 1.11028^T	25.4	67.0	2.64
Flagellimonas sp. C4^T	17.3	55.7	3.22
Flagellimonas sp. GZD32^T	25.3	70.0	2.77
